# Preventing Phosphorylation of Sterol Regulatory Element-Binding Protein 1a by MAP-Kinases Protects Mice from Fatty Liver and Visceral Obesity

**DOI:** 10.1371/journal.pone.0032609

**Published:** 2012-02-27

**Authors:** Jorg Kotzka, Birgit Knebel, Jutta Haas, Lorena Kremer, Sylvia Jacob, Sonja Hartwig, Ulrike Nitzgen, Dirk Muller–Wieland

**Affiliations:** 1 Institute of Clinical Biochemistry and Pathobiochemistry, German Diabetes Center at the Heinrich-Heine-University Duesseldorf, Leibniz Center for Diabetes Research, Duesseldorf, Germany; 2 Institute for Diabetes Research, Asklepios Clinic St. Georg, Department of General Internal Medicine, Medical Faculty of Semmelweis University, Hamburg, Germany; Institut Pluridisciplinaire Hubert Curien, France

## Abstract

The transcription factor sterol regulatory element binding protein (SREBP)-1a plays a pivotal role in lipid metabolism. Using the SREBP-1a expressing human hepatoma cell line HepG2 we have shown previously that human SREBP-1a is phosphorylated at serine 117 by ERK-mitogen-activated protein kinases (MAPK). Using a combination of cell biology and protein chemistry approach we show that SREBP-1a is also target of other MAPK-families, i.e. c-JUN N-terminal protein kinases (JNK) or p38 stress activated MAP kinases. Serine 117 is also the major phosphorylation site in SREBP-1a for JNK. In contrast to that the major phosphorylation sites of p38 MAPK family are serine 63 and threonine 426. Functional analyses reveal that phosphorylation of SREBP-1a does not alter protein/DNA interaction. The identified phosphorylation sites are specific for both kinase families also in cellular context. To provide direct evidence that phosphorylation of SREBP-1a is a regulatory principle of biological and clinical relevance, we generated transgenic mice expressing mature transcriptionally active N-terminal domain of human SREBP–1a variant lacking all identified phosphorylaton sites designed as alb-SREBP-1aΔP and wild type SREBP-1a designed as alb-SREBP-1a liver specific under control of the albumin promoter and a liver specific enhancer. In contrast to alb-SREBP–1a mice the phosphorylation–deficient mice develop no enlarged fatty livers under normocaloric conditions. Phenotypical examination reveales a massive accumulation of adipose tissue in alb-SREBP-1a but not in the phosphorylation deficient alb-SREBP-1aΔP mice. Moreover, preventing phosphorylation of SREBP-1a protects mice also from dyslipidemia. In conclusion, phosphorylation of SREBP-1a by ERK, JNK and p38 MAPK-families resembles a biological principle and plays a significant role, *in vivo*.

## Introduction

Sterol regulatory element binding proteins (SREBPs) are the predominant transcription factors controlling the synthesis of cholesterol and fatty acids in liver [1]. The family of SREBPs encompasses essentially two isoforms, SREBP–1 and SREBP–2, encoded by the corresponding genes SREBF-1 and SREBF-2 [2], [3]. In contrast to SREBF–2, SREBF–1 is transcribed into two major splice variants, SREBP-1a and SREBP-1c, differing in the first exon of the mature protein [2], [3]. Several lines of evidence indicate that SREBP–2 is the master regulator of cholesterol synthesis, whereas the isoform SREBP–1c controls the synthesis of fatty acids and is unique in the SREBP family in the regulation of lipogenesis by carbohydrates. However, the isoform SREBP–1a can control both pathways of lipid synthesis to a main degree [1], [3]–[9]. The different functions for the SREBP-1 isoforms are mainly thought to be casued by the different length of the N-terminal transactivation domain. Whereas this domain of SREBP-2 and SREBP-1a has a comparable length the respective N-Terminal domain of SREBP-1c is much shorter and a much weaker transcriptional activator. [10]. So SREBP-1a, -1c and-2 may have different specifics to the members of the family of binding and integrator proteins. Whereas SREBP-1a and -2 is active to a similar degree on genes with a promoter with the classical sre-1-element SREBP-1c is not. On the other hand SREBP-1a and SREBP-1c act comparable on genes with a promoter containing E-box element, but not SREBP-2 [8], [9], [11], [12].

Furthermore the SREBP-1 isoforms differ in the expresson levels, e.g. transcripts of SREBP-1c are approximately 10 fold higher abundant in liver as SREBP-1a [3], [13]. SREBP-1c is thought to be a basal transcription factor and SREBP-1a that has a much stronger transactivation activity is thought to be responsible for regulation upon physiological demands [5].

One essential feature of SREBPs is that they are transcribed and translated into inactive precursor molecules which are embedded in the membrane of the cellular endoplasmatic reticulum. The composition of this pool still remains unclear and an accumulation of either SREBP isoform, even if the expression rates are reduced cannot be excluded. To generate the transcriptional active domain, SREBPs are released by a complex two-step sequential proteolytic machinery initiated by cholesterol depletion of the cell. Only the mature forms of SREBPs translocate in the nucleus and activate target genes [14]. Beside proteolytic cleavage, which controls the abundance the transcriptional activity of SREBP-1a can be directly modified by phosphorylation and we have demonstrated that trans-activity of the mature N-terminal domain of SREBPs is regulated directly by extra cellular stimuli, e.g. by hormones such as insulin [15]–[20]. Moreover, in these studies, we have shown that the N-terminal domains of SREBP-1a, SREBP-1c, and SREBP-2 are direct substrates of the extracellular signal-regulated kinase (ERK) subfamily of mitogen-activated protein kinases (MAPK). This mechanism might place SREBP-1a as a gene regulatory point of convergence for inflammation, insulin resistance, obesity and intracellular lipid accumulation, since hormones, cytokines, drugs, and free fatty acids are able to activate SREBPs via MAPK cascades [18].

It is well known that other MAPK cascades, like JNK (c-JUN N-terminal protein kinase) as well as p38 MAP kinases, target transcription factors directly by phosphorylation, thereby altering their transactivity, leading to changes of cellular gene regulatory networks [21]. However, it is still unclear whether these MAP kinases, which are triggered by inflammatory and stress signals including cytokines can also regulate directly intracellular lipid homeostasis. This could be a missing link between inflammation and alterations in lipid accumulation, which are also frequently associated with insulin resistance, obesity, and an increased cardiovascular risk.

Since SREBP-1a is the master regulator of both pathways, i.e. synthesis of fatty acids as well as cholesterol, we had the hypothesis, that SREBP-1a is phosphorylated by JNK and p38 MAPK thereby altering transcriptional activity and cellular lipid metabolism. Accordingly in this report we show, that SREBP–1a is directly phosphorylated by members of stress kinases families, i.e JNK and p38 MAPK. Using protein chemistry methodology we identify the major kinase specific phosphorylation. In HepG2 cells we have shown, that identified phosphorylation sites are also targets in cellular context. Furthermore, to study physiological and clinical impact of SREBP-1a phosphorylation, we generated mouse models that overexpress the transcriptional active wild type domain of SREBP-1a or a construct in which all identifed MAPK phosphorylation were destroyed by mutation in liver. Using these transgenic mouse models we provide first evidence that phosphorylation of SREBP–1a at these site appears to be of physiological relevance.

## Results

### Identification of stress activated MAP kinases specific phosphorylation sites in SREBP-1a

**Figure 1:**
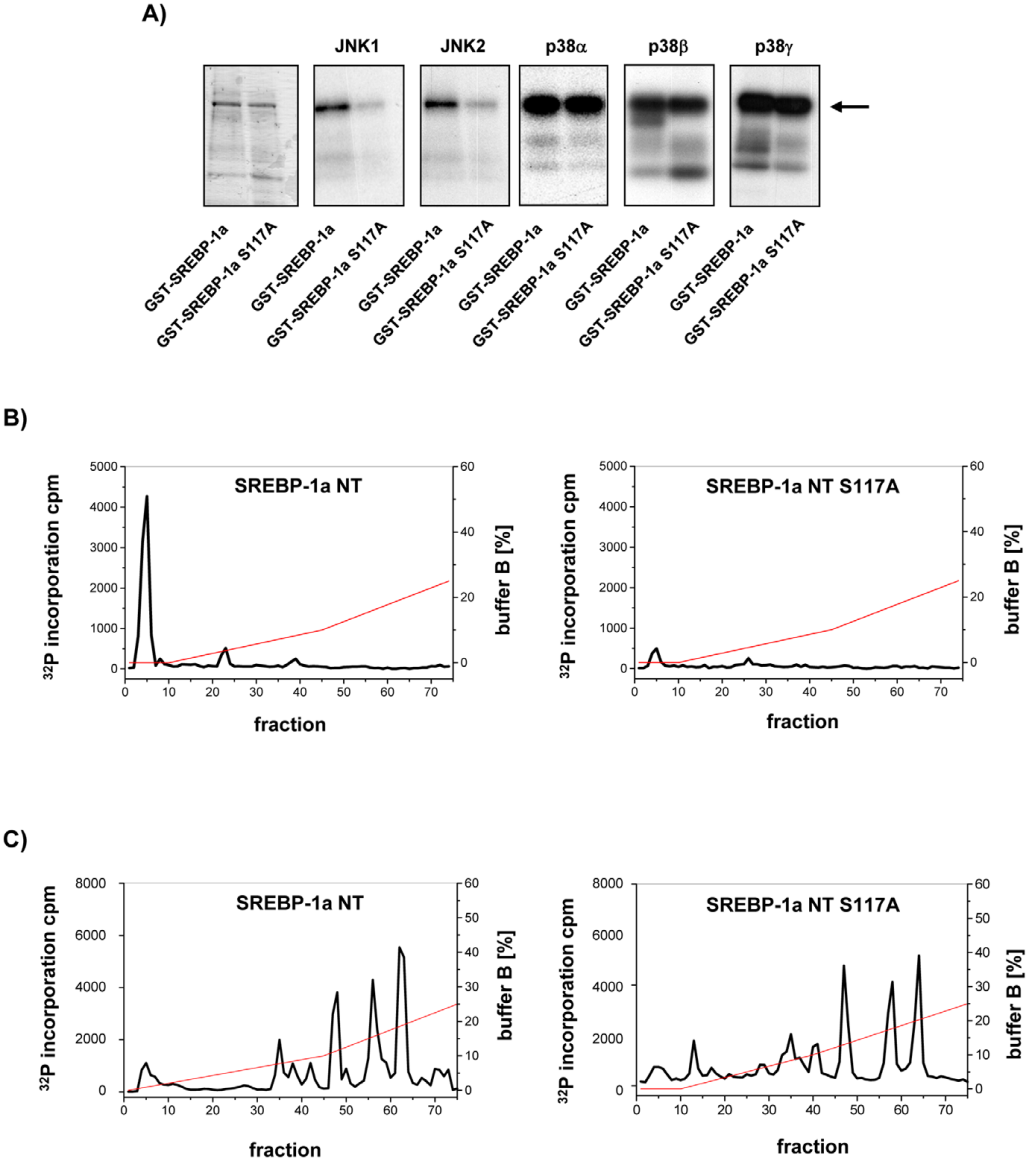
Identification of S117 as major JNK related phosphorylation site in SREBP-1a. (**A**) 10 µg GST-SREBP-1a-NT and GST-SREBP-1a-NT S117A were phosphorylated by activated recombinant JNK1 or JNK2 (40 ng/µg protein) or recombinant p38 isoforms p38α, p38β, p38γ (40 ng/µg protein) *in vitro* and separated by 10% SDS-PAGE. A coomassie blue stained gel (left), autoradiography of SDS-PAGE of JNK1, JNK2, p38α, p38β or p38γ phosphorylation is shown. The arrow marks band of SREBP-1a. The excised radioactive slices of GST-SREBP-1a-NT or GST-SREBP-1a-NT S117A phosphorylated by JNK1 (**B**) or phosphorylated by p38α (**C**) were digested by trypsin and the resulting peptides were subjected to anion exchange chromatography. The graph shows the elution fraction plotted against the incorporated radioactivity. Reactions performed are described under “Materials and Methods”.

Previously we have shown that the N-terminal domain of human SREBP-1a is a substrate for ERK kinase and identified S117 as the major phosphorylation site [16]. Here we investigated if SREBP-1a is also substrate of JNK and p38 MAPK families. To identify the major related phosphorylation sites in we incubated the N-terminal domain of SREBP-1a (SREBP-1a-NT) as purified recombinant GST fusion protein with activated purified recombinant JNK1 and JNK2 or p38α, ß and γ (Figure 1A). The results showed that SREBP-1a-NT was also an efficient substrate for all kinases with an average of 0.9 mole phosphates per mole protein for JNK isoforms and 1.5 mole phosphates per mole protein for the p38 isoforms. To test whether major ERK site S117A is also the target of JNK or p38, the S117A mutant of GST-SREBP-1a-NT was phosphorylated by JNK1, JNK2 or p38α, ß and γ. The phosphorylation efficiency of JNK1 or JNK2 on SREBP-1a S117A was reduced by 90%, whereas phosphorylation by the p38 kinase isoforms were not altered compared to wild type (Figure 1A). This indicates that JNK1 and 2 but not p38 phosphorylate SREBP at position S117. Anion exchange HPLC maps of trypsin-digested SREBP-1a-NT GST fusion proteins phosphorylated by JNK show one predominant peak that was abolished in the profile performed with GST-SREBP-1a-NT S117A (see Fig. 1B) confirming S117 as the major phosphorylation site for JNK in SREBP-1a.

The profile of SREBP-1a-NT phosphorylated by p38 was completely different and more complex (Figure 1C). Using the GST-SREBP-1a-NT wild type and S117A revealed similar patterns. The results showed that SREBP-1a was very efficiently phosphorylated by all p38 kinases but the identified major phosphorylation site for ERK and JNK, namely S117, was not the target of p38 phosphorylation in SREBP-1a.

**Figure 2:**
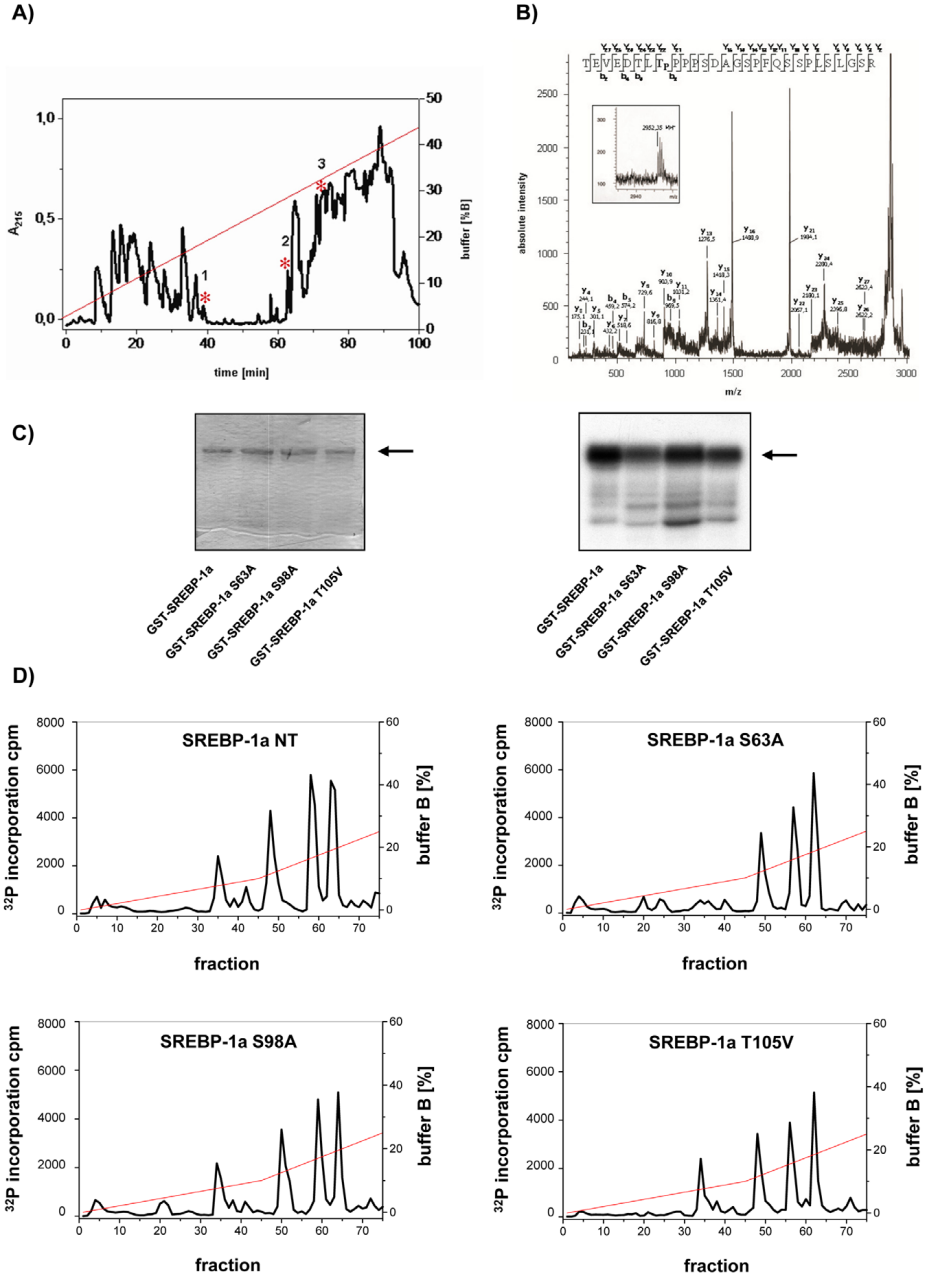
Identification of p38 specific phosphorylation sites in SREBP-1a-NT. (**A**) 500 µg GST-SREBP-1a-NT were phosphorylated by p38α (40 ng/µg protein). Following 10% SDS-PAGE and in-gel trypsin digestion of phosphorylated SREBP-1a-NT, the resulting peptides were fractionated by reverse-phase HPLC on a C18 column. Peptides were eluted with a 0–95% linear gradient of acetonitrile in 0.1% trifluoroacetic acid. The eluate was monitored by UV absorbance at 215 nm. Radioactive fractions are marked with asterisks. (**B**) The HPLC fraction 1, containing radiolabeled peptide, was analyzed by MALDI-MS in the linear positive-ion mode and lead to identification of a (M+H)^+^ peptide having a *m*/*z* of 2952.35 Da. MALDI-PSD analyses using this peptide ion as mass parent ion. Fragmentation mass spectrum of (M+H)^+^ peptide with identified internal b- and y-fragment ions are designated. The interpretation of PSD fragment data allowed identification of the above-designated peptide (TEVEDTL**T** ^*^PPPSDAGSPFQSSPLSLGSR) (aa 419–446) with phosphorylated T426. (**C**) 10 µg GST-SREBP-1a-NT and GST-SREBP-1a-NT S63A, GST-SREBP-1a-NT S98A or GST-SREBP-1a-NT T105V were phosphorylated by activated recombinant p38 isoforms p38α, p38β, p38γ (40 ng/µg protein) *in vitro* and separated by 10% SDS-PAGE. A coomassie blue stained gel (left-hand side) and an autoradiography (right-hand side) of SDS-PAGE of p38α, p38β or p38γ phosphorylation are shown. The arrow marks the band of SREBP-1a. (**D**) The excised radioactive slices of GST-SREBP-1a-NT, GST-SREBP-1a-NT S63A, GST-SREBP-1a-NT S98A, or GST-SREBP-1a-NT T105V phosphorylated by p38α were digested by trypsin and the resulting peptides were subjected to anion exchange chromatography. The graph shows the elution fraction plotted against the incorporated radioactivity. Reactions performed are described under “Materials and Methods”.

In order to identify the major p38 MAP kinases specific phosphorylation sites in SREBP-1a we analyzed *in vitro* phosphorylated radiolabelled GST-SREBP-1a-NT by reversed phase mass spectrometry. The radiolabelled peak 1 phosphopeptide detected in an UV elution profile (Figure 2 A) was identified as human SREBP-1 aa 419–446 in which T426 was phosphorylated by mass spectrometry (Figure 2B) whereas peak 2 and peak 3 could not be assigned directly. Edman degradation assays identified peak 2 and peak 3 as identical peptides representing the N-terminal part of mature SREBP-1a (aa 1–108). This peptide contains three typical consensus sequences for MAPK (PXS/TP) phosphorylation sites i.e. S63, S98 and T105. GST fusion proteins containing the corresponding mutants i.e. S63A, S98A and T105V were analyzed by *in vitro* phosphorylation assays (Figure 2C) and by anion exchange HPLC of the trypsin-digested proteins (Figure 2D). Compared to wild type SREBP-1a (Figure 2B) the first peak of SREBP-1a-NT profile is lost by S63A, whereas neither S98A nor T105A altered the elution profiles (Figure 2D). This provided strong evidence that S63 is a second major phosphorylation site for p38 in SREBP-1a beside T426.

**Figure 3:**
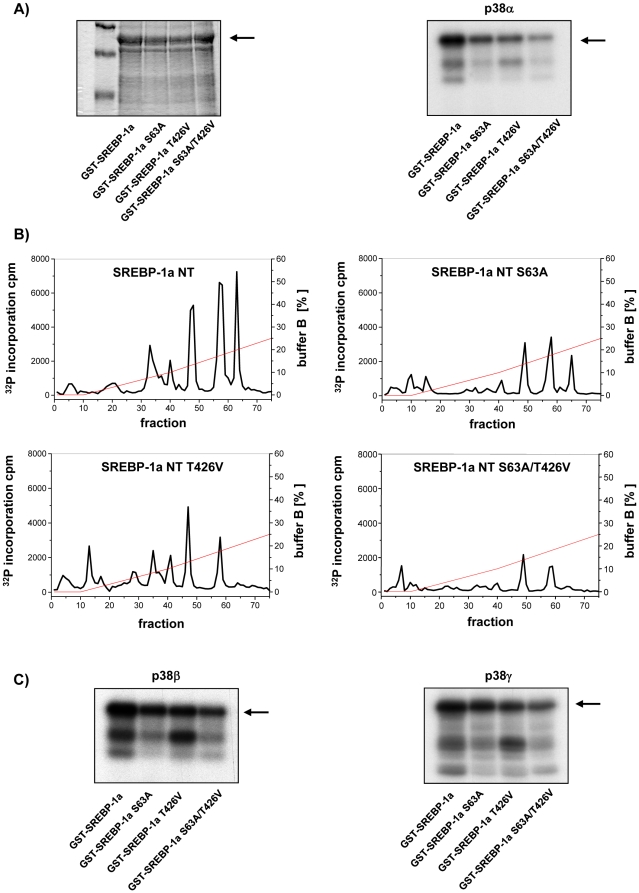
Verification of p38 specific phosphorylation sites in SREBP-1a-NT. (**A**) Coomassie brilliant blue stained SDS-PAGE of GST-SREBP-1a-NT and each of mutated SREBP-1a-NT phosphorylated by activated recombinant p38α (40 ng/µg protein). (**B**) Autoradiography of SDS-PAGE of GST-SREBP-1a-NT fusion protein, single mutated forms S63A, T426V or double mutant GST-SREBP-1a-NT S63A/T426V phosphorylated by activated recombinant p38α. Excised radioactive slices of p38α phosphorylated recombinant proteins were trypsin-digested and the resulting peptides GST-SREBP-1a-NT, S63A, T426V or double mutant S63A/T426V, as indicated in the figure, were subjected to anion exchange chromatography. Elution was performed with a KH_2_PO_4_ pH 4 buffer gradient. Reactions performed are described under “Materials and Methods”. (**C**) Autoradiography of SDS-PAGE of GST-SREBP-1a-NT fusion protein, single mutated forms S63A, T426V or double mutant GST-SREBP-1a-NT S63A/T426V phosphorylated by activated recombinant p38β or p38γ (40 ng/µg protein).

MAPK p38α *in vitro* phosphorylation assay using single mutants SREBP-1a-NT S63A and SREBP-1a-NT T426V as well as the double mutant, i.e. SREBP-1a-NT S63A/T426V supported the findings. Compared to wild type SREBP-1a-NT phosphate incorporation was reduced by approximately 60% for each single mutation and approximately 80% in the double mutant S63A/T426V (Figure 3A). This was confirmed by anion exchange chromatography of each p38α phosphorylated trypsin-digested SREBP-1a-NT. Each single mutant and the double mutant S63A/T426V were lacking any significant radioactive fraction (Figure 3B). Thus, we conclude that S63 and T426 are the predominant phosphorylation sites of p38α in SREBP-1a. Phosphorylation assays of SREBP-1a-NT or mutated SREBP-1a-NT (S63A, T426V and S63A/T426V) using p38β or p38γ (Figure 3C) revealed that S63 and T426 were not a specific feature of p38α isoform rather than the p38 family. The reduction of phosphate incorporation in the mutated proteins was comparable to the reduction obtained with p38α (compare Figure 3C with Figure 3A).

### JNK and p38 MAPK phosphorylation of SREBP-1a did not intefere with protein/DNA interaction

**Figure 4:**
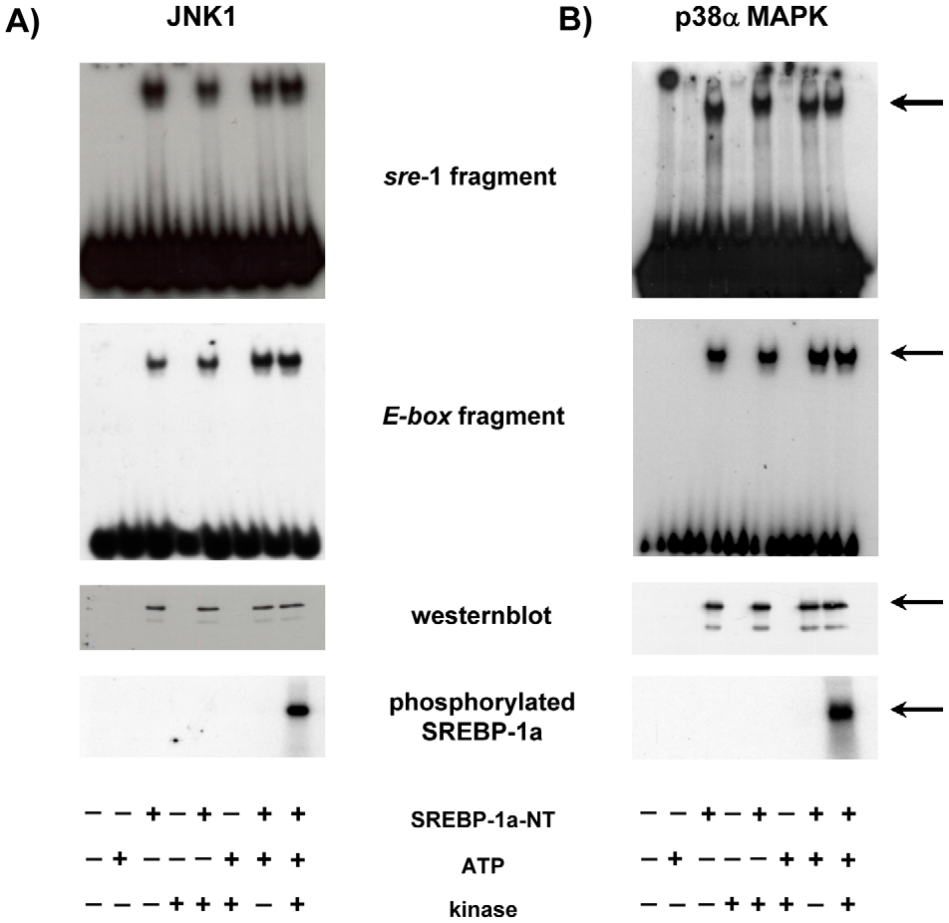
Effect of phosphorylation on DNA binding and acitvity of SREBP-1a *in vitro*. (**A**) His-SREBP-1a-NT fusion protein was incubated with or without JNK1 (40 ng/µg protein) as indicated. An EMSA of His-SREBP-1a-NT incubated with sre-1 fragment (upper panel) or E-box fragment (lower panel) is shown. To confirm equal loading, western-blot analyses with monoclonal anti-HisG-HRP antibody was performed. To control phosphorylation efficiency a JNK1 kinase assay with (γ^32^P) ATP was performed and dried SDS-PAGE was exposed to X-ray film. For further details see “Materials and Methods”. (**B**) His-SREBP-1a-NT fusion protein was incubated with or without p38α (40 ng/µg protein) as indicated. An EMSA of His-SREBP-1a-NT incubated with sre-1 fragment (upper panel)or E-box fragment (lower panel) is shown. To confirm equal loading, western-blot analysis with monoclonal anti-HisG-HRP antibody were performed. To control phosphorylation efficiency a p38α kinase assay with (γ^32^P) ATP was performed and dried SDS-PAGE was exposed to X-ray film. For further details see “Materials and Methods”.

Phosphorylation can influence protein/DNA interaction. Therefore we used *in vitro* transcribed and translated His-SREBP-1a-NT and *in vitro* kinase assays to monitor the effect of phosphorylation on protein/DNA interaction. We incubated *in vitro* transcribed and translated His-SREBP-1a-NT without or with JNK1 or p38α and subsequently analyzed the protein/DNA interaction by EMSA (Figure 4A, B). SREBP-1a-NT interacted with sre-1 as well as E-box motif to a similar degree. Phosphorylation of SREBP-1a-NT affected protein/DNA interaction neither by JNK1, nor by p38α. This is in accordance to the observation that ERK phosphorylation of SREBP-1a at S117 does not influence protein/DNA interaction [16].

### JNK and p38 MAPK target the identified phosphorylation sites of SREBP-1a in intact cells

**Figure 5:**
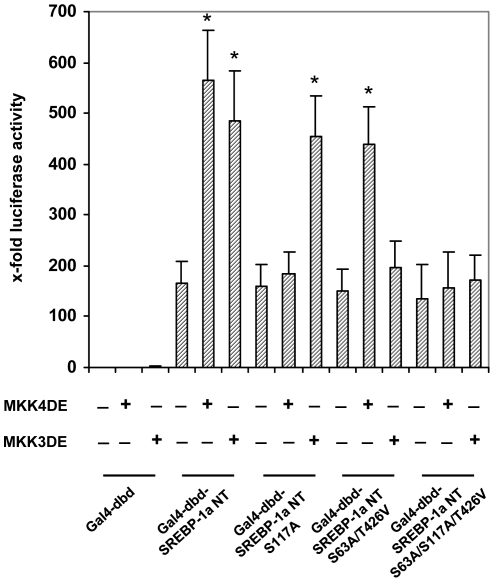
Effect of phosphorylation on DNA binding and acitvity of SREBP-1a. HepG2 cells were transiently transfected with pG5-luc (0.5 µg/well) and pFA-SREBP-1a-NT or the mutants S117A, S63A/T426V, S63A/S117A/T426V (25 ng/well) in the presence 25 ng/well MKK3DE and MKK4DE, respectively as indicated. Cells were maintained for 16 h before harvesting and analyses of dual luciferase activity. Transfection efficiency was controlled by co-transfection of pRL(-mcs)-vector (0.1 µg/well). Promoter strength of the LDL receptor gene is represented by the relative luciferase activities. Results are given as means (±S.D.) of five independent experiments, each performed in triplicate (p<0.01).

To test the relevance of identified phoshorylation sites for JNK as well as p38 MAPK more specificily in cellular context we performed promoter reporter gene assays recruiting the heterologous Gal4-system and specific kinases for the MAP kinases cascades (Figure 5). In this assay, SREBP-1a-NT and the mutated forms were expressed as fusion proteins with the DNA binding domain of yeast Gal4 (aa 1–147). The latter domain alone doesn't have any trans-activity per se. Accordingly, activities detected reflect trans-activities of SREBP-1a-NT or its mutants fused to the yeast Gal4 domain. To activate JNK and p38 signalling selectively in Hep G2 cells, the constitutively active upstream activators for JNK, i.e. MKK4DE, or p38, i.e. MKK3DE, were used. To monitor the specific initiation of JNK and p38 signaling to SREBP-1a in HepG2 cells MKK4DE or MKK3DE were cotransfected with wildtype pFA SREBP 1a NT or respective phosphorylation site mutants. Basal trans-activities of all mutated forms of SREBP-1a-NT were comparable to wildtype. Constitutive active MKK4DE or MKK3DE synergistically elevated trans-activity of SREBP-1a-NT by about 3-fold. Transfection of activated MKK3 or MKK4 showed that S117A specifically interferes with MKK4 signaling, whereas S63A and T426V abolished MKK3 signaling. Accordingly the triple mutant (SREBP-1a-NT S63A/S117A/T426A) failed to be activated by both kinase pathways. These results clearly show that SREBP-1a was a specific target for JNK and p38 activation, since the specific upstream kinases trigger kinase signaling to the specificphosphorylation sites.

### Relevance of SREBP-1a phosphorylation *in vivo*

**Figure 6:**
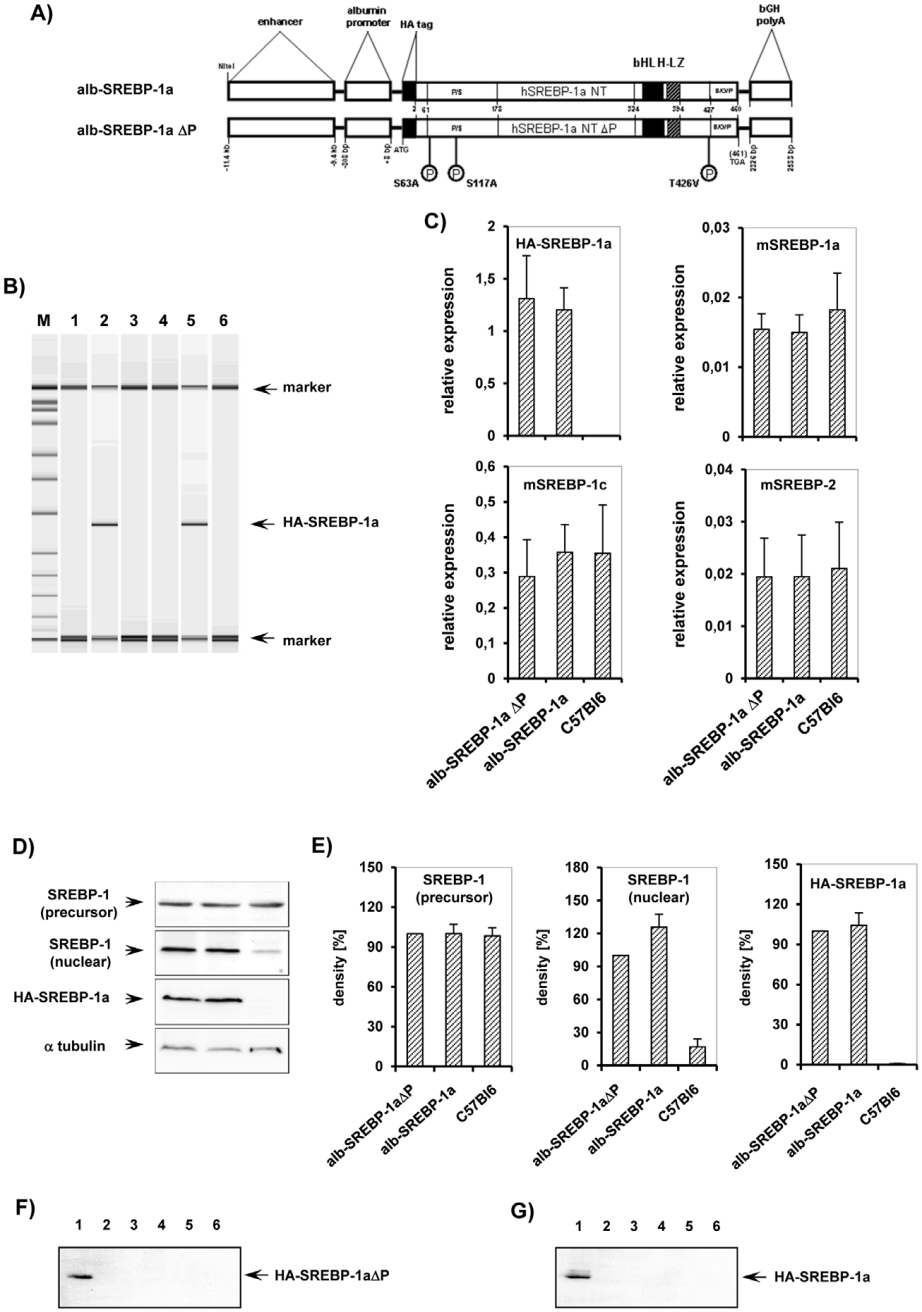
Liver specific overexpression of HA-SREBP-1aΔP and HA-SREBP-1a *in vivo*. (**A**) Scheme of the DNA constructs used to generate transgenic mice. The transcriptional active N-terminal domain of the phosphorylation mutant of the human SREBP-1a, SREBP-1aΔP, or the human SREBP-1a gene were inserted into a vector construct containing the mouse albumin promoter and a liver specific enhancer element next to a HA-Tag and a polyadenylation site. Constructs only differ in three aminoacids, i.e. the MAPK phosphorylation sites S63, S117 and T426 that have been mutated to S63A, S117A and T426V as indicated in the scheme. (**B**) Verification of transgene insertion into genomic DNA by PCR. M: marker, genomic DNA of lane 1: C57Bl6, 2: alb-SREBP-1aΔP, 4: C57Bl6, 5: alb-SREBP-1a, lane 3 and 6: no template controls. (**C**) Validation of transgene expression on mRNA level by RT-PCR. RNA extracted from snapp frozen liver biopsies from male alb-SREBP-1aΔP, alb-SREBP-1a or C57Bl6 mice were analyzed by RT-PCR with transgene human HA-SREBP-1a, mouse SREBP-1a, mouse SREBP-1c and mouse SREBP-2 specific primers and probe. The relative RNA amount shown in arbitrary units was calculated and plotted ± SD. Graphs represent data from ten male mice per genotype, each analyzed in triplicate (p<0.01). (**D**) Verification of transgene expression on protein level in liver. Protein extracts of snap frozen liver biopsies from lane 1: SREBP-1aΔP, 2: alb-SREBP-1a, and 3: C57Bl6 mice were separated by SDS-PAGE and blotted on nitrocellulose membrane. Membranes were probes with SREBP-1 specific antibodies to determine SREBP-1 precursor protein (K10, SantaCruz) and total nuclear SREBP-1 (H160, SantaCruz) contend. To determine the HA-tag of the transgene construct membrabes were probed with HA-specific antibody. For normalizing blots were probed with α-tubulin antibody. A representative experiment is shown. (E) Graphs show densitometry evaluation for SREBP-1 precursor, nuclear SREBP-1 contend and the transgenic HA-SREBP-1a construct of n = 5 independent experiments with the levels determined for SREBP-1aΔP set as 100%. Tissue specific expression of alb-SREBP-1aΔP (**F**) and alb-SREBP-1a (**G**). Protein extracts of lane 1: liver, 2: pancreas, 3: heart, 4: kidney, 5: small intestine and 6: skeletal muscle, were separated by SDS-PAGE, blotted and probed with HA-specific antibody. A representative experiment is shown. The arrow indicates HA-tagged SREBP-1a.

To investigate the biological relevance of MAPK related phosphorylation of human SREBP 1a *in vivo*, we generated transgenic mice which express selectively in liver the mutant variant of human SREBP-1a lacking all identified major phosphorylation sites, designated as SREBP-1aΔP (Fig. 6A). In addition we generated transgenic mice which express liver specifically the wild type of human SREBP-1a to a similar degree. Our intent was to study the role of SREBP 1a phosphorylation in liver for the phenotype of mice. Taken into consideration the complex and highly regulated releasing cascade of mature SREBP-1a protein from ER anchoured precursor proteins and to circumvent secondary cholesterol feedback effects [4] we used the N–terminal transcriptional active domain. To facilitate detection a HA-tag was added and for liver specific expression the albumin promotor and a liver specific enhancer was chosen. The HA-tag did not affect phosphorylation, trans-activation or protein/DNA interaction of the N–terminal transcriptional active domain of SREBP-1a *in vitro* (data not shown). The mice were bred on C57Bl6 background which also served as global controls throughout the study. Viability, fertility or breeding efficiency and nesting behaviour of the transgenic animals was not affected and the observed life span was not reduced.

The presence of the transgenes was monitored by genotyping of the intersection of albumin promoter and SREBP-1a coding sequence (Figure 6B). As mouse strains might have different expression levels of the transgenes due to physical integration of the construct in the genome, we decided to follow strains per genotype whose expression of transgene had a similar degree (Figure 6C). In our SREBP-1a overexpressing mouse models the expression of the differential spliced SREBP-1a isoform SREBP-1c or SREBP-2 was not altered (Figure 6C). Moreover the expression of endogenous SREBP-1a determined with mouse specific primers and probe was not altered in the transgenic mice, too (Figure 6C). Western blot analyses (Figure 6D) of liver extracts for SREBP-1 protein indicate no alteration in the SREBP-1 precursor protein fraction in SREBP-1aΔP, SREBP-1a and C57Bl6 mice. The total contend of nuclear SREBP-1 in liver extracts shown in western blot analyses with an SREBP-1 specific antibody detecting human and mouse SREBP-1 is increased in the transgenes compared to C57Bl6. Using HA-tag specific antibody revealed the expression of the respective transgenes at the protein level to the same degree, too (Figure 6D, 6E). Western blot analyses with extracts of liver, pancreas, heart, kidney, small intestine or skeletal muscle tissue proved that transgenes are expressed solely in liver of alb-SREBP-1aΔP (Figure 6F) or alb-SREBP-1a (Figure 6G) animals.

To determine the functionality of the liverspecific overexpression of the human transcriptional active domain of SREBP-1a and the impact of SREBP-1aΔP the gene expression of key metabolic enzymes was analyzed in liver of these animals (Table 1) as a functional proof of principle. The expression rates of genes involved in lipid metabolism like FAS, SCD or GPAT and the rate limiting gene in cholesterol metabolism, HMG-CoAR were at least doubled in alb-SREBP-1 aΔP mice compared to C57Bl6 but increased more than 10 fold in alb-SREBP-1a mice compared to C57BL6 mice. Genes involved in lipid transport as LDLR or ABCA1 were increased 2.5-fold in alb-SREBP-1 aΔP mice and increased more than five- fold in alb-SREBP-1a mice. Interstingly MTTP expression was not significantly altered in alb-SREBP-1 aΔP but significantly increased in alb-SREBP-1 a mice. In SREBP-1 aΔP PEPCK expression was elevated six-fold compared to a fifteen-fold increase in alb-SREBP-1 a mice. There was an approximately doubling of GLUT-2 expression in alb-SREBP-1a mice whereas the increase in alb-SREBP-1aΔP was not significantly altered to C57Bl6. Rate limiting metabolic genes as liver specific pyruvate kinase or malic enzyme expression were significantly increased in alb-SREBP-1aΔP compared to C57Bl6 but this again does not reach the increase observed in alb-SREBP-1a animals. Taken together compared to C57Bl6 the effect of the phosphorylation deficient SREBP-1aΔP is a clear inducibility of gene expression but the effect is by far not as pronounced as in the wildtype alb-SREBP-1a mice. Furhtermore for all genes investigated a significant increase of expression can be obtained also to SREBP-1aΔP mice.

**Table 1:** Gene expression of lipid metabolic genes in livers of C57Bl6 and alb-SREBP-1c.

	C57Bl6	alb-SREBP-1aΔP	alb-SREBP-1a
**FAS**	7.88±3.14	37.49±10.81^**^	272.90±94.05^**‡‡^
**SCD1**	152.30±78.34	302.76±70,11^**^	2145.72±498.32^**‡‡^
**GPAT**	0.75±0.40	1.88±0.81^**^	11.93±2.89^**‡‡^
**HMG-CoAR**	1.29±0.37	2.39±0.37^**^	18.99±6.41^**‡‡^
**LDLR**	0.35±0.05	0.90±0.32^**^	2.07±0.59^**‡‡^
**ABCA1**	0.44±0.03	1.12±0.31^**^	2.33±0.61^**‡‡^
**MTTP**	13.65±5.03	18.73±6.60	48.77±15.66^**‡‡^
**PEPCK**	4.06±2.46	23.61±10.02^**^	61.03±20.82^**‡‡^
**GLUT2**	17.39±3.41	21.18±6.12	38.03±6,5^**‡‡^
**pyruvate kinase**	58.75±8.05	54.30±12.41	111.08±34.81^**‡‡^
**malic enzyme**	0.01±0.002	0.01±0.003	0.02±0.001^**‡‡^

The hepatic expression level of genes were determined by RT-PCR (n = 20 each). The relative RNA amount shown in arbitrary units was calculated and plotted ± S.D. (C57Bl6 vs. alb-SREBP-1a or alb-SREBP-1aΔP mice: **p<0.001; alb-SREBP-1aΔP vs. alb-SREBP-1a mice: ‡‡p<0.001.)

### Mutation of phosphorylation sites in SREBP-1a prevents excessive weight gain under normocaloric conditions

**Figure 7:**
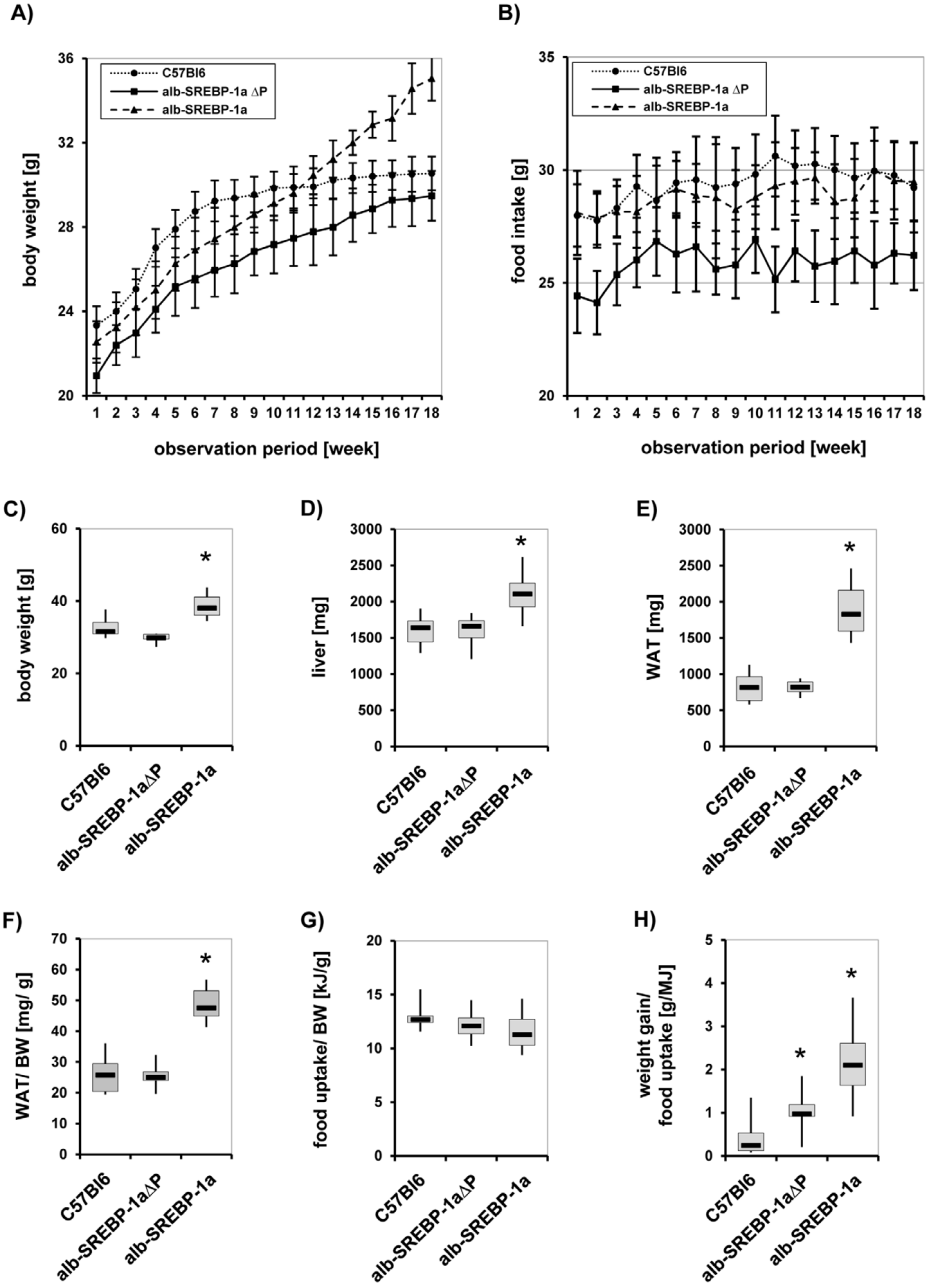
Weight gain and Food intake of C57Bl6, alb-SREBP-1aΔP and alb-SREBP-1a transgenic animals. Male C57Bl6, alb-SREBP-1aΔP and alb-SREBP-1a mice (n = 20 per genotype) were housed as groups of four under standard conditions with unlimited access to water and regular chow (13.0 MJ/kg: 53% carbohydrates, 11% fat, 36% protein). Weight gain (**A**) and Food intake (**B**) were measured once a week starting at weaning and monitored for an observation period of 18 weeks. Body weight (**C**), liver weight ((**D**) and WAT weight (**E**) were determined at sacrification. WAT contend per body weight (**F**), food uptake per body weight and (**G**) weight gain per food uptake (**H**) were determined in each group of mice. Data are given as means including standard deviation (±SD).

Transgenic male alb-SREBP-1aΔP and alb-SREBP-1a as well as control C57Bl6 mice were kept in groups of four under standardized conditions starting with normocaloric standard diet after weaning at the age of 6 weeks for an observation period of further 18 weeks. Monitoring the weight gain revealed that C57Bl6 mice continously gain weight until they reach a plateau up to the 15^th^ week (Figure 7A). Initially alb-SREBP-1aΔP mice were approximately 10% smaller than C57Bl6 and had a weight gain of only 30% of C57Bl6 mice with a parallel growth curve. At the age of 6 weeks the alb-SREBP-1a mice were comparable to C57Bl6 mice, but the weight gain of alb-SREBP-1a mice persists continously. A post-study observation at age of 30 week indicated their maximum of weight gain with a mean weight of 40 g (data not shown).

Food intake of C57Bl6 and alb-SREBP-1a mice was not different whereas alb-SREBP-1aΔP mice consumed approximately 20% less (Figure 7B). At 24 weeks body weight of alb-SREBP-1aΔP mice was like C57Bl6 wereas alb-SREBP-1a were significantly higher (Figure 7C). This was also observed for liver weight and white adipose tissue (Figure 7D, E). The ammount of white adipose tissue per body weight was nearly doubled in alb-SREBP-1a whereas again alb-SREBP-1aΔP and C57 were identical (Figure 7F). In relation to body weight food uptake was comparable in both models and controls (Figure 7G). In contrast, weight gain per food intake disclosed that the amount of food consumed by alb-SREBP-1aΔP was significantly higher than C57Bl6. More impressively this was calculated for alb-SREBP-1a mice (Figure 7H). Taken together, preventing phophorylation of the overexpressed transgene SREBP-1a by mutation of the phosphorylation sites resulted in reduced weight gain per food consumed compared to overexpression of the functional wild-type SREBP-1a.

### Preventing phosphorylation of SREBP-1a protects from fatty liver

**Figure 8:**
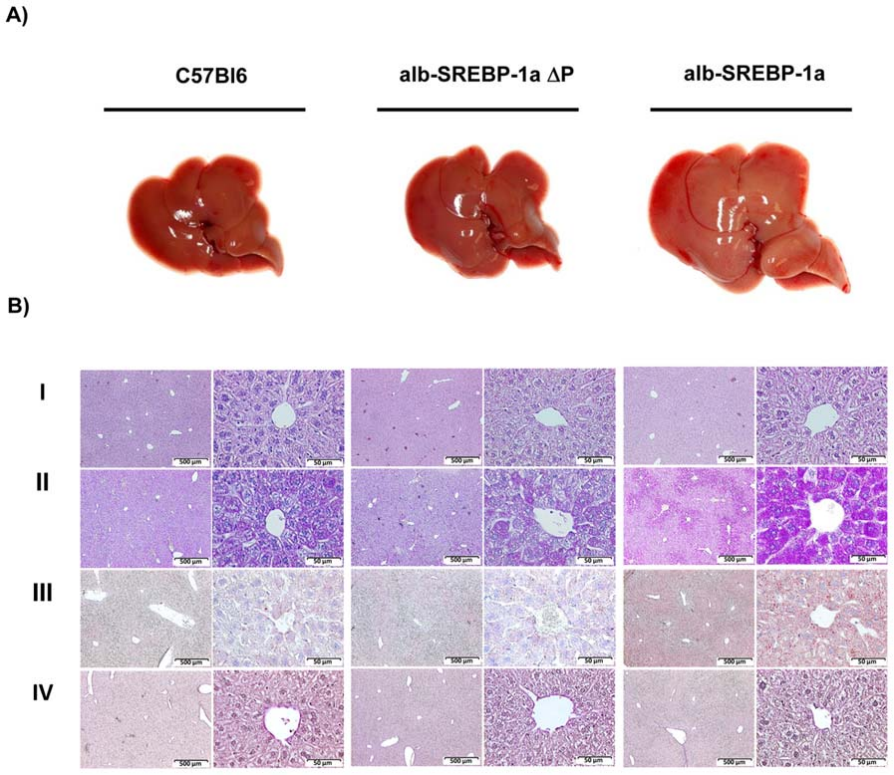
Macroscopic and histological comparison of livers from C57Bl6, alb-SREBP-1aΔP and alb-SREBP-1a mice. Male mice of each genotype (C57Bl6, alb-SREBP1aΔP, alb-SREBP-1a (n = 20 per genotype)) were housed as groups of 4 under standard conditions with unlimited access to water and regular chow (13.0 MJ/kg: 53% carbohydrates, 11% fat, 36% protein). (**A**) Livers of a C57Bl6 mouse (left), alb-SREBP-1aΔP (middle) or alb-SREBP-1a (right). All photographs were taken with the same magnification. (**B**) Liver tissue of the Lobus caudatus, Lobus sinister- and Lobus dexter lateralis were fixed in 4% paraformaldehyd/PBS and embedded in paraffin with automated standard histological procedures. (I) Standard hematoxylin and eosin staining was performed on 3 µm deparaffinized sections. (II) PAS staining was performed to determine glycogen contend. (III) The tissues were also used for cryofixation and Oil-red-O staining was used for lipid visualization. (IV) Fibers and extra cellular matrix were visualized using the “van Gierson kit” to determine tissue integrity. The overview magnification is 1∶10 and details are shown in 1∶80 magnification.

Macroscopic liver examination of transgenic and control mice at the age of 24 weeks revealed that the appearance of alb-SREBP-1aΔP livers were slightly enlarged, but in alb-SREBP-1a mice the mean liver size were raised about 25% and colouring was slightly pale (Figure 8A). For further morphological analyses tissues of the Lobus caudatus, Lobus sinister or dexter lateralis were taken (Figure 8B). In C57Bl6 and alb-SREBP-1aΔP morphological intact parenchym with dense cytoplasm, clear nucleus, eosinophilic nucleii and basophile euchromatin can be observed. In contrast in alb-SREBP-1a mice the general impression of the liver tissue of was more dimorph, cytoplasma was less dense with more vacuoles and enlarged cell volume. C57Bl6 and alb-SREBP-1aΔP show similar glycogen granula with slightly perilobular concentration. Glycogen content in alb-SREBP-1a mice was higher and mainly centered arround the lobus. In some cells it presents as granula or not related to distinct structures of the cytosol. In alb-SREBP-1aΔP mice accumulation of visible lipid droplets mainly located around the nucleus was slightly higher than in C57Bl6 mice. Contrary, alb-SREBP-1a mice showed strong lipid accumulation centered around the portal vein spanning whole areas of the cytosol. In cells with highest ectopic lipid accumulation yet no signs of cytotoxicity, i.e. degradation of the nuclear structures, were observed. No gross alterations of connective tissue, collagene fibers or fibrocytes were detected in C57Bl6 and alb-SREBP-1aΔP mice indicating no signs for fibrosis. In alb-SREBP-1a mice the matrix was also without any specific alterations. In all cases no Ito cells, infiltrating monocytes or other inflammation markers were detected.

### Preventing phosphorylation of SREBP-1a protects from visceral obesity

**Figure 9:**
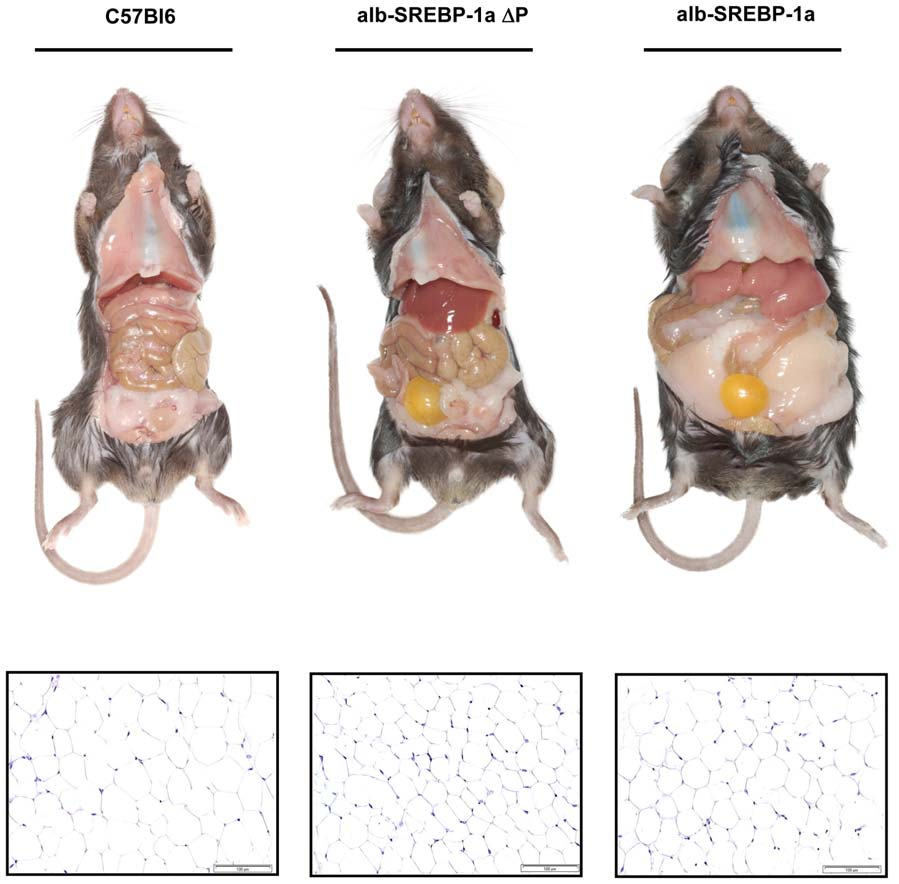
Macroscopic and comparison of C57Bl6, alb-SREBP-1aΔP and alb-SREBP-1a mice. (**A**) Male C57Bl6, alb-SREBP1aΔP and alb-SREBP-1a (n = 20 per genotype) were housed as groups of four under standard isocaloric conditions with unlimited access to water and regular chow (13.0 MJ/kg: 53% carbohydrates, 11% fat, 36% protein). Panels show a ventral view of C57Bl6, alb-SREBP-1aΔP and alb-SREBP1a mice. (**B**) For histological overview standard hematoxylin and eosin staining was performed on 3 µm deparaffinized sections of WAT. All photographs are in same magnification.

Macroscopic examination of mice at the age of 24 weeks revealed that the phenotype of alb-SREBP-1aΔP mice was similar to C57Bl6 mice (Figure 9A). In contrast to that, in alb-SREBP-1a mice the epididymale and inguinal fat mass was massively increased leading to a “potato-shaped” appearance of the animals. In phosphorylation deficient alb-SREBP-1aΔP mice no excess fat mass could be determined. Histological sections of the adipose tissue revealed adipocyte hyperplasie but no hypertropy and also no signs of macrophage infiltration (Figure 9B). So, SREBP-1a specifically expressed in liver results not only in the development of enlarged fatty livers but also has a massive impact on whole body fat mass. This alteration of body composition was abolished by preventing SREBP-1a from phosphorylation.

### Systemic influence of SREBP-1a phosphorylation

As expected from the macroscopic examination determination of body weight, liver weight, and weight of visceral fat indicated that alb-SREBP-1a animals had significantly increased values of serum parameters including dyslipidemia and insulin resistance, whereas the alb-SREBP-1aΔP mice were more comparable to wildtype C57Bl6 (Table 2). Triglyceride levels but not serum cholesterol were massively increased in alb-SREBP-1a mice whereas the phosphorylation deficient alb-SREBP-1aΔP showed a slightly reduced TG level as C57Bl6 mice. Free fatty acid (FFA) levels in serum showed that in alb-SREBP-1aΔP mice an elevation by 50% compared to C57BL6 can be determined and alb-SREBP-1a mice have a circa threefold higher value as C57Bl6. According to this only the alb-SREBP-1a mice had significantly elevated liver function tests, i.e. GPT/ALT and GOT/AST, respectively. Leptin levels were elevated only in alb-SREBP-1a mice in accordance to the increased fat mass. Blood glucose and insulin were increased in alb-SREBP-1a mice, whereas in alb-SREBP-1aΔP mice soley insulin was modestly elevated.

**Table 2:** Basal serum parameters at the state of scarification.

	C57Bl6	alb-SREBP-1aΔP	alb-SREBP-1a
**cholesterol [mg/dl]**	104.62±15.29	83.39±14.09	93.47±10.15
**triglceride [mg/l]**	121.10±27.84	94.89±24.42	235.27±39.78^**‡‡^
**FFA serum [g/l]**	0.89±0.09	1.39±0.14^**^	2.53±0.21^**‡‡^
**TFA liver [mg/g tissue]**	24.9±4.2	37.0±2.9^**^	97.2±11.9^**‡‡^
**blood glucose [mmol/l]**	7.77±0.89	7.16±0.77	10.90±2.49^**^
**insulin [ng/ml]**	0.92±0.41	1.36±1.32	4.09±1.36^**^
**leptin [ng/ml]**	15.81±5.71	20.11±8.05	44.39±6.78^**^
**ALT [U/l]**	34.05±10.69	42.72±17.15	55.67±26.86^**^
**AST [U/l]**	36.38±16.58	46.61±13.38	60.07±18.67^**^

Parameters were measured in C57Bl6, alb-SREBP1aΔP and alb-SREBP-1a mice housed as groups of 4 under standard conditions with unlimited access to water and regular chow (13.0 MJ/kg: 53% carbohydrates, 11% fat, 36% protein) at 24 weeks of age. Data are given as mean ± SD (n = 20). (**p<0.01: alb-SREBP-1aΔP or alb-SREBP-1a vs. C57Bl6; ‡‡p<0.01: alb-SREBP-1aΔP vs. alb-SREBP-1a).

**Figure 10:**
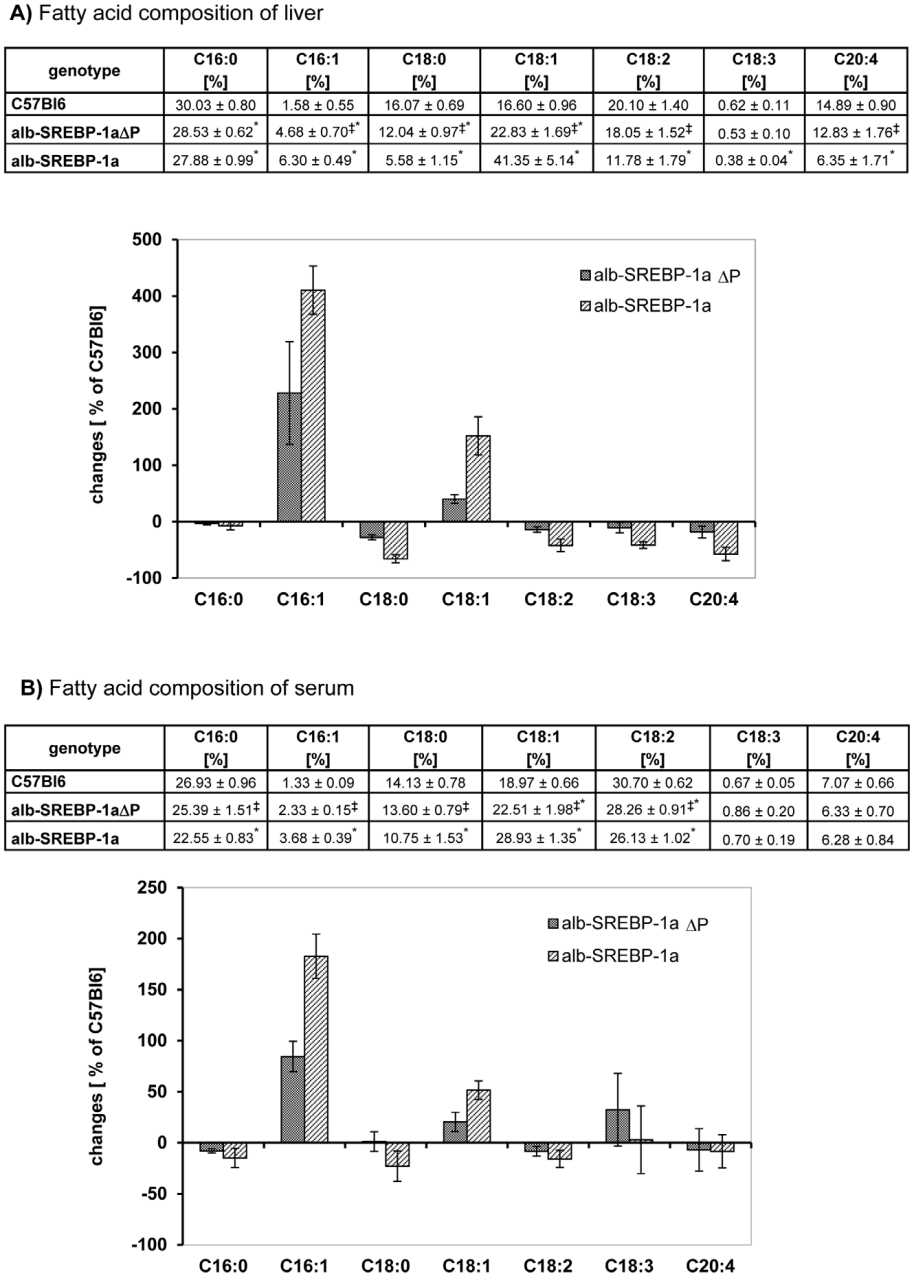
Phosphorylation of SREBP-1a influences fatty acid composition of liver and serum. In liver tissues the composition total fatty acid (TFA) (**A**) and in serum samples of the same animals the composition of free fatty acid (FFA) (**B**) of C57Bl6, alb-SREBP1aΔP as well as alb-SREBP-1a transgenic animals was determined by GC analyses. Data were calculated as percent of in TFA or FFA. The graphs indicate the percental difference the values determined for alb-SREBP-1aΔP and alb-SREBP-1a mice to C57Bl6 mice. Data are given as means including standard deviation (±SD) of (n = 20) replicates per genotype (*p<0.01: alb-SREBP-1aΔP or alb-SREBP-1a vs. C57Bl6; ‡p<0.01: alb-SREBP-1aΔP vs. alb-SREBP-1a).

As expected the content of total fatty acid (TFA) in liver of alb-SREBP-1a mice was approximately fourfold higher as in liver of C57Bl6 mice. In contrast the phosphorylation deficient mice were mostly protected against lipid accumulation and show a 1.5 fold- increase. Detailed analyses of fatty acid composition in liver tissues and serum were performed (Figure 10). In liver the content of saturated FA, i.e. C16:0 was unaltered and C18:0 was slightly reduced in phosphorylation deficient mice alb-SREBP-1aΔP but massively reduced alb-SREBP1a mice in comparison to C57Bl6. The level of mono unsaturated FA C16:1 was increased three fold in alb-SREBP-1aΔP and four fold alb-SREBP-1a mice. C18:1 showed an 1.5 fold- increase in alb-SREBP-1aΔP and a 2.5 fold- increase alb-SREBP-1a mice. Polyunsaturated fatty acids C18:2, C18:3 and C20:4 contend showed a genotypespecific reduction in the transgenic mouse models which was most prominent in alb-SREBP-1a mice whereas alb-SREBP-1aΔP mice were more comparable to C57Bl6. Compared to the levels determined for C57Bl6, there is a pronounced increase in C16:1 and C18:1 for alb-SREBP-1aΔP that is further doubled in alb-SREBP-1a mice. Also the reduced levels for the further FA observed in alb-SREBP-1aΔP were nearly doubled in alb-SREBP-1a mice (Figure 10A). In serum the FFA pattern of mice is essentially comparable to liver, but the differences are not that pronounced. Also the differences in relation to C57Bl6 when significant, again show a pattern with the largest differences observed for alb-SREBP-1a mice and the alb-SREBP-1aΔP beeing intermediate to C57BL6 and alb-SREBP-1a mice (Figure 10B).

### Phosphorylation of SREBP-1a influences insulin sensitivity

**Figure 11:**
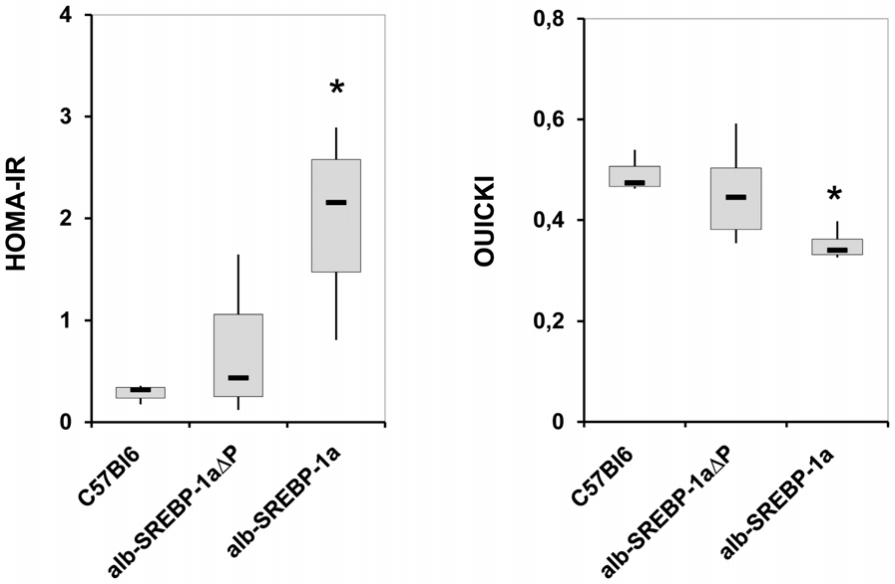
Phosphorylation of SREBP-1a influences systemic insulin sensitivity. Surrogate indexes were calculated from fasting blood glucose and plasma insulin concentrations as follows: QUICKI = 1/(log(I0)+log(G0)), where I0 is fasting insulin (µU/ml) and G0 is fasting glucose (mg/dl); and HOMA-IR = (G0 * I0)/22.5, with fasting glucose expressed as mmol/l and fasting insulin expressed as µU/ml. Data are given as box plot analyses (n = 20). ANOVA significance p<0.05 is indicated by an asterisks.

The mice overexpressing functional SREBP-1a in liver developed a fatty liver, but the phenotype is abrogated if the main phosphorylation sites in SREBP-1a were mutated as in alb-SREBP-1aΔP mice. Excess intracellular lipid accumulation in hepatocytes impairs liver functionality and insulin sensitivity. Caculating of HOMA-IR index as surogate for insulin resistance indicated that alb-SREBP 1aΔP mice have a comparable level to C57Bl6, but the variation within this genotype is broarder. The HOMA-IR index of the alb-SREBP 1a mice indicated the beginning of insulin resistance. QUICKI as surogate for insulin sensitivity indicated for alb-SREBP 1aΔP that the insulin sensitivity was in normal range but with larger varaition that the controls. In contrast for alb-SREBP 1a mice the insulin sensitivity and ß-cell failure were significantly pronounced (Figure 11). So also for these parameters the alb-SREBP-1aΔP show values intermediate to C57BL6 and alb-SREBP-1a mice.

### Phosphorylation of SREBP-1a reduced serum cytokine pattern

**Figure 12:**
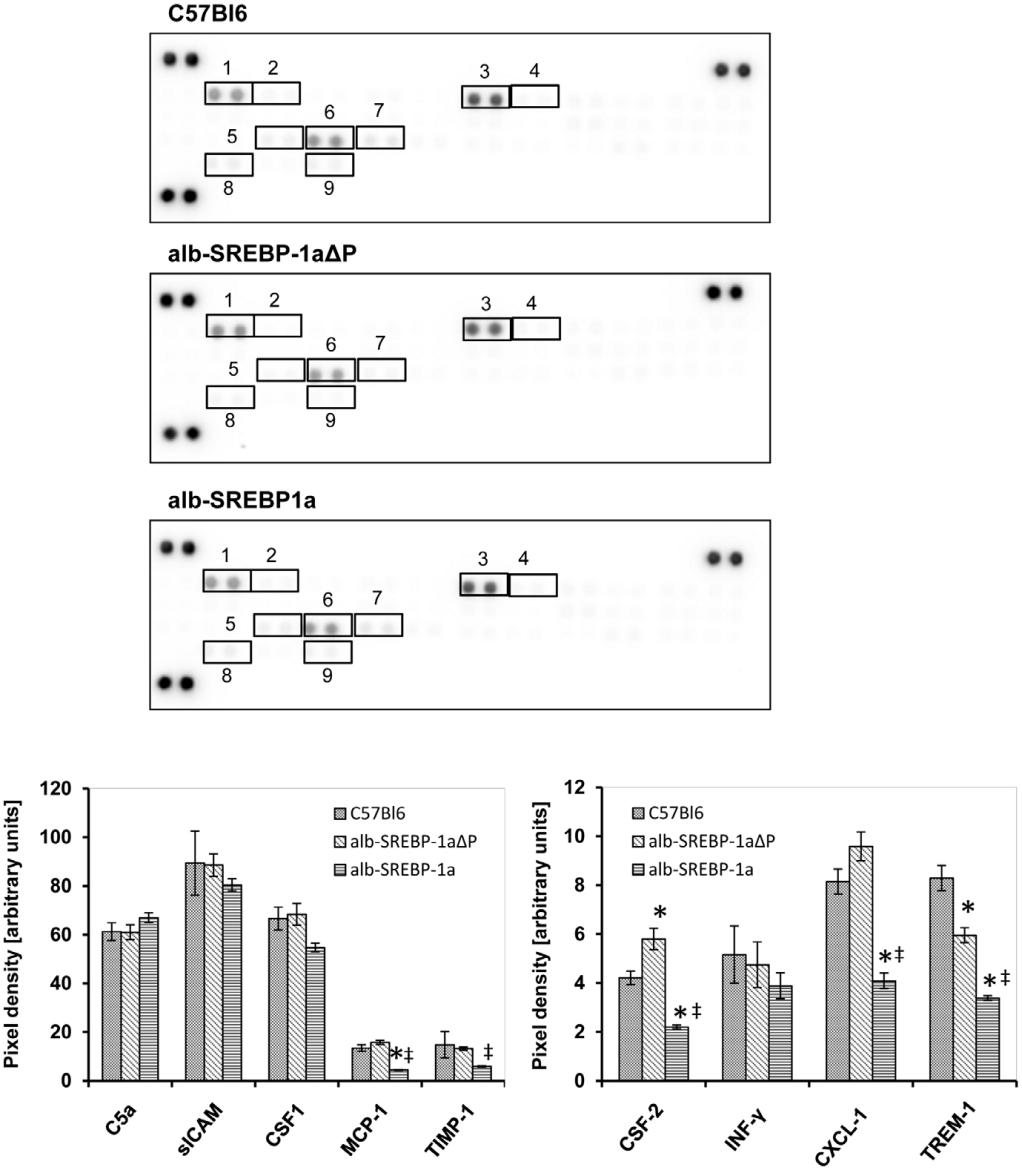
Phosphorylation of SREBP-1a influences serum cytokine profile. The cytokine content in serum was analyzed using the Proteome Profiler™; R&D Systems, (Abingdon, UK). Spot intensities were normalized to background and positive controls set to 100% intensity. Presented numbers on membranes mark targets as follows: (1) C5a; (2) CSF-3; (3) sICAM; (4) INF-γ; (5) IL-12-p70; (6) CXCL-1; (7) CSF-1; (8) MCP-1; (9) Timp-1; (10) Trem-1. Abundance of: CXCL13, CSF-2, CCL-1, CCL-11, IL1-α, IL1-ß, IL-1ra, IL-2, IL-3, IL-4, IL-5, IL-6, IL-7, IL-10, IL-13, IL-16, IL-17, IL-23, IL-27, CXCL-10, CXCL-11, MCP-5, CXCL-9, CCR-1a, CCL-4, CCL-2, CCL5, CXCL-12, CCL-17 or TNF-α was not detected in serum. (1) CSF-3; (2) GM-CSF CSF-2; (3) CCL-1; (4) sICAM -; (5) IL-1ra; (6) IL-6; (7) CXCL-10; (8) CXCL-1; (9) CSF-1; (10) MCP-1; (11) CXCL-9; (12) MCP-5; (13) CCR-1a; (14) CXCL-2; (15) CCL-5; (16) Timp-1. Abundance of: CXCL-13, C5a, CCL-11, IFN-γ, IL1-α, IL1-ß, IL-2, IL-3, IL-4, IL-5, IL-7, IL-10, IL-13, IL-12-p70, IL-16, IL-17, IL-23, IL-27, CXCL-11, CCL-4, CXCL-12, CCL-17, TNF-α or Trem-1 was not detected. Data are given as means ± S.D. (n = 6, each) of normalized intensity. Significance was calculated by 2-way ANOVA. (C57Bl6 vs. alb-SREBP-1a or alb-SREBP-1aΔP mice: *p<0.001; alb-SREBP-1aΔP vs. alb-SREBP-1a mice: ‡p<0.001.)

Wether obesity or ectopic hepatic lipid accumulation are associated to inflammation is still under debate [22]–[24]. To determine wether the lipid accumulation in liver and the vast increase of adipose tissue un the alb-SREBP-1a mice affets inflammatory parameters and wether the phosphorylation deficent mutant has an impact on inflammation, a set of 40 chemokines and cytokines was analyzed in serum of control and transgenic animals (Figure 12). In C57Bl6 chemokines, i.e. C5a, sICAM, CSF-1, CSF-2, MCP-1, INF-γ, CCL-1, CCL-11, CXCL-1, Timp-1 and Trem-1, but no proinflammatory cytokines could be determined (Figure 12).

No genotype specific cyokine pattern can be detected in serum of alb-SREBP-1a and alb-SREBP-1aΔP mice, nevertheless the abundance of some cytokines is altered. Again the alb-SREBP-1aΔP show a cytokine profile more comparable but not totally identical to C57Bl6. An increase in CSF-2 and CXCL-1 and a reduction in TREM-1 can be otained. In contrast, significant alterations in the alb-SREBP-1a mice is the reduced abundance of the respective chemokines exept for C5a and sICAM where no alteration can be obtained.

## Discussion

SREBP-1a has been identified as a connective link at the gene regulatory level integrating signaling of various hormones, cytokines, and metabolites [17]. Recruitment of MAPK cascades is a general mechanism for a rapid gene regulatory answer of cells to environmental stimuli by setting the activation state of different transcription factors. JNK and p38 MAPKs target transcription factors directly by phosphorylation thereby altering their transactivity leading to changes of cellular gene regulatory networks [21].

Here, we provide direct evidence that SREBP–1a is a selectively phosphorylated target of JNK and p38 signaling cascades and might link MAPK cascades to lipid metabolism.

MAPK cascades are the paradigm of activiation by sequential phosphorylation with high target substrate specificity. Previously, we have shown that the SREBP transcription factors are targets for intracellular signaling pathways [16]–[20]. We could show that SREBPs are substrates for ERK-MAPK and identified 432 and 455 as major phosphorylation in SREBP-2 and serine 117 in SREBP-1a [16], [19]. Here we demonstrate that serine 117 in SREBP-1a is also the major phosphorylation for JNK site but does not play a role for p38 MAPK signaling instead S63 and T426, indicating selective phosphorylation of SREBP-1a by different MAPK-families. JNK shares the major phosphorylation site S117 in SREBP1-a with ERK, a phenomenon which is also observed in the regulation of other transcription factors [25]–[27]. On the other hand differential targeting of transciption factors by MAPK is a general phenomenon, shown for example at ETS-domain transcription factor Elk-1, c-Myc, and nuclear receptors [25]–[27]. MAPK phosphorylation sites are designated by the consensus sites S/TP [28], PXS/TP [29]–[31] or PXXS/TP [31]. The specificity of the different members of the MAPK family and of the different isoforms of p38 MAPK is provided by a docking motif usually composed of three domains: the basic region, the LXL motif, and the hydrophobic region [32]. The hydrophobic region seems to be of particular importance for the determination of the substrate specificity for p38 MAPK [33], [34]. In the case of SREBP-1a one can postulate that the S63 is the main phosphorylation site as adjacent to the LXL motive (LSL: aa 33–36) a complete PXS/TP motive, i.e. PASP (aa 61–64), is present whereas at position T426 the LXL motive (LSL: aa 385–387) is followed by an incomplete S/TP motive, i.e. TP (aa 426–427), that might function as a weaker phosphorylation target for p38. The identified phosphorylation sites differ in their consensus sequence, i.e. the JNK-specific site has the PXXSP motive, whereas the p38-specific sites are PXSP for S63 and TP for T426. [31]. As many potential substrates contain these motifs, it is important to provide further specificity determinants to direct individual kinases towards the correct target. Interaction with specific docking proteins and specific recruitment of substrates via the docking domains of the MAPK exists. These docking domains can enhance the efficiency of phosphorylation, and to generate signaling specificity. This modular signal transmission in the cell can be seen as the basis for an economic utilization of signaling components to implement cellular response to altered environmental conditions with limited signaling molecules. In the case of SREBP-1a the activation of the protein following different stimuli seems to be necessary as an increase of SREBP-1a driven genes can be involved in the response to altered nutrient composition, growth requirements or cellular stress. So a preferential phosphorylation of distinct sites depending on the cellular demands can be modified according to the signal. Therefore phosphorylation of SREBP–1a appears to be a regulatory step linking stress signals with metabolic events, i.e. cellular lipid metabolism in the case of SREBP-1.

Phosphorylation of SREBP-1a might also affect other posttranslational modifications. The work of Sundqvist et al. [35] showed that the amino acid T426 and the adjacent S430 in mature SREBP-1a is involved in the ubiquitin-dependent protein degradation and mutation to alanine of these sites increased protein stability. This phosphorylation dependent degradation was initiated by phosphorylation of mature SREBP-1a through the protein kinase GSK-3. Up to now there is no evidence of phosphorylation S63 and S117 in mature SREBP-1a on protein stability. Yang and coworkers demonstrated that phosphorylation of the transcription factor Elk-1 is not only differentially by various MAPK cascades but activation is associated with a rapid loss of SUMO modification [36]. This loss was related to increased transcriptional activity. Interactions between phosphorylation and SUMOylation have been described in SREBP-2 [37] and might also interfere with transcriptional activation or protein stability. Mutation of the phosphorylation site in SREBP-2 increases the SUMOylation and therefore it was concluded, that hormone-induced phosphorylation reduces SUMOylation [37]. This will result in a complex regulative interplay. The SUMO sites K464 in SREBP-2 is in direct proximity of the phosphorylation site S432 and S455, which we have identified previously [19]. In the case of SREBP-1a, one of the SUMO sites, i.e. K123, is in close proximity to S117. In addition K418 was identified as a further SUMO site in SREBP-1a, but until today it had not been further investigated because the adjacent phosphorylation site was unknown. In this respect it is interesting to note, that the p38 site T426 is identical to the the GSK-3 site and T430 appears to be in close proximity in SREBP-1a, making an interaction likely. Different signalling cascades can affect SREBP-1 phosphorylation and interfere with the molecular function. For example the SREBP-1 isoform SREBP-1c has recently been shown to be phosphorylated via AMPK at S372 or SIK at S265, S266 and S325, respectively [38], [39] Although mechanisms involved do not directly increase the transcriptional activity of the molecule but affect localization and stability and there is no overlapp to the MAPK phosphorylation sites identified in the present study, finally these modifications also alter the gene expression rate of SREBP-1c target genes. Further studies would have to explore the interaction of these regulatory possibilities.

In general the N-terminal transcriptional active domain of SREBP-1a has a basal transcriptional activity. In cell culture experiments increasing the amount of SREBP-1a by overexpression is sufficient to induce transcription. This basal transcriptional activation can be synergstically increased if there is a further induction i.e. a hormonal stimulus to initiate the signalling cascades to phosphorylation [15]–[17], [20]. This phosphorylation solely affects the N-terminal transcriptional active domain of SREBP-1a, and not the precursor protein [15]. Mechanistically the effect on increasing transcriptional activity by phosphorylation gets obvious as the identified S63 and S117 are in the acid domain of SREBP-1a (amino acids 1–61). Transactivity will be significantly enhanced by adding further negative charges, i.e. phosphate incorporation. The identified N-terminal phosphorylation sites (S63, S117) are located in the acidic domain of SREBP-1a whereas T426 is near the dimerization as well as the DNA binding bHLH-LZ domain (amino acids 324–394) that exhibits the highest sequence homology in the SREBP family. The position of the latter phosphorylation site resembles the position of the phosphorylation sites for ERK-MAPK which we had identified previously in SREBP-2 [19]. Deduced from the position, phosphorylation of T426 in SREBP-1a might influence dimerization or DNA binding. However, we show here that phosphorylation on these sites didn't affect the bindinding of SREBP-1 to both known core promoter elements, i.e. sre-1 or E-box. On the other hand, transactivity of SREBP-1a is stimulated by phosphorylation and hampered by the phosphorylation deficient mutants accordingly.

The protein pool in the nuclear envelop seems to provide sufficient mature SREBP–1a for posttranslational modification. Previously we have shown that phosphorylation of SREBP-1a does not interfere with mRNA expression, maturation or protein/DNA interaction of SREBP-1a, but is related to ERK-MAPK pathway inducible increase of e.g. gene expression of SREBP-1a target genes like the LDLR and central genes of lipid metabolism in cell culture models [20].

The limitation of cell culture models always is that they can't answer the question whether a mechanism identified has biological importance and possibly allows the explanation of a pathophysiological process. To adress the potential biological importance of SREBP-1a phosphorylation *in vivo* we have created mouse models which express specifically in liver either the mature form of SREBP–1aΔP, which is deficient in all identified MAP kinase phosphorylation sites, or the wild type SREBP–1a. Due to the complex cholesterol dependant feedback regulation of SREBP–1a secretion from membranes and the fact that cellular cholesterol overload restrains the SCAP–SREBP–1 complexes to the membranes, we overexpressed the transcriptional active N–terminal domain directly, circumventing secondary effects due to the complex and highly regulated releasing cascade of mature SREBP–1a [14]. The key advantage of the utilization of the albumin promoter in our system is that it turned out that the animals develop a pathological phenotype already under normocaloric nutrition. There is no need of further physiological provocation by feeding the animals a specific i.e. high fat diet to induce a phenotype. This is of central importance as we intended to monitor mainly the effect of SREBP-1a phosphorylation and not the abundance of the molecule. The effect of the posttranslational phosphorylation process otherwise might be masked behind effects triggered by affluent food supply or specific food components like increased transcription of the transgene that might be induced in the PEPCK-SREBP-1a model [40]. Detailed comparison of our animals shows that although both transgenic models overexpress the transcriptional active N-terminal domain of SREBP-1a in the liver to a comparable degree, overexpressing of the functional SREBP-1a wild type shows a vast accumulation of lipids in the liver including obesity and insulin resistance, whereas the phosphorylation deficient alb–SREBP–1aΔP mice is protected. The hepatic gene expression of key SREBP-1a target genes in the alb-SREBP-1a and alb-SREBP1aΔP mice indicated the functional overexpression of the respective molecules. Overexpression of the N-terminal active domain of SREBP-1a in mouse liver alters the regulation of SREBP-1a target genes. This was comparable to the gene expresion alterations reported [40], Compared to C57Bl6 the effect in the phosphorylation deficient alb-SREBP-1aΔP mice is a clear inducibility of gene expression but the effect is by far not as pronounced as in the wildtype alb-SREBP-1a mice. So the phosphorylation deficient mutant does not act as a dominant negative manner but the synergistical transcriptional activity induced by SREBP-1a overexpression and phosphorylation is lost in these animals.

Clinical studies have shown that the intracellular amount of lipid in liver is associated with insulin resistance. Therefore syndromes like the non alcoholic fatty liver disease appears to be a hepatic manifestation of insulin resistance or the metabolic syndrome [41]–[43]. To further get insight to the physiological basis of the observed phenotypes the status of insulin resistance and insulin sensitivity of the animals was of interest. To get some evidence it is feasible to use the surrogate indexes of insulin resistance and sensitivity, i.e. HOMA-IR and QUICKI, developed for humans which have sucessfully adopted to mice [44]–[47]. Altough blood glucose levels are unaffected in the alb-SREBP-1aΔP mice and only slightly elevated in alb-SREBP-1a animals the indices in relation to insulin levels clearly show a beginning of insulin resistance in SREBP-1a wild type mice compared to phosphorylation deficient mice, which appears to be primarily related to altered liver metabolism and the degree of hepatic lipid accumulation. So all serum parameters indicate that the alb-SREBP–1aΔP mice are protected of the fatty liver phenotype compared to the alb-SREBP–1a mice, which develop fatty liver, obesity, hypertriglyeridemia, and insulin resistance.

The pattern of circulating cytokines are a indicator for the physiological condition of the liver. There is ongoing discussion of the role of inflammation in the pathogenesis of obesity and fatty [22]–[24] Neither alb-SREBP–1aΔP mice nor alb-SREBP–1a aminals show signs of increased secretion of cytokines involved in the inflammatory systems. The alterations in the investigated mice of serum cytokines affects mainly cytokines involved in cellular regeneration and maintenance processes. This could indicate that compensatory regeneration processes are active to cope with the stress induced by the lipid accumlation in alb-SREBP-1a mice. The missing indication of inflammation could be a hint that at the present stadium of lipid accumulation in hepatocytes the liver has not converted to a pathological condition or promoting apoptosis, yet.

Taken together, we have shown that SREBP-1a is phosphorylated by JNK and p38 MAPK at different specific sites. Furthermore, direct evidence is provided that phosphorylation does not influence DNA binding but phosphorylation of the identified sites plays a role in stress mediated effects on LDLR promoter activity or activation and therefore affects target gene expression. These data further support our concept that, besides sterol-dependent cleavage, SREBPs are regulated by phosphorylation affecting their trans-activity by various MAPK cascades leading to changes of cellular gene regulatory networks [18]–[20].

Our investigations implicate that in liver phosphorylation of SREBP-1a has biological implications on the development of fatty liver and massive weight gain. This is impressively demonstrated by the phosphorylation deficient SREBP-1aΔP animals which are are protected against all facets of the phenotype including lipid accumulation, visceral adipositas and signs of persisting insulin reristance. Phosphorylation of SREBP-1 also has clinical implications as we have recently reported a rare heterozygous mutation in a patient with severe dyslipidemia hampering specifically phosphorylation of Erk- and JNK-MAPK specific S117 in SREBP-1a [48]. These findings strongly support the biological relevance of the posttranslational modification of SREBP–1a.

## Materials and Methods

### Plasmid constructs

N-terminal domain of human sterol regulatory element binding protein (SREBP)-1a was inserted in frame to glutathione-S-transferase (GST) into pGEX 3X to generate SREBP-1a-NT GST-fusion protein as previously described [16]. Generation of SREBP-1a-NT S117A was described elsewhere [16]. The construction of the mutated forms of SREBP-1a-NT (S63A, S98A, T105V, T426V, S63A/T426V and S63A/S117A/T426V) was produced by site-directed mutagenesis using the Quick Change Mutagenesis Kit (Stratagene, Amsterdam, Netherland) according to manufacturer's recommendations. Expression vectors containing SREBP-1a-NT were generated by cloning of corresponding fragments from pGEX plasmids into pcDNA3.1/HisA (Invitrogen, Karlsruhe, Germany). To construct Gal4-SREBP-1a-NT, Gal4-SREBP-1a-NT S117A, Gal4-SREBP-1a-NT S63A/T426V and Gal4-SREBP-1a-NT S63A/S117A/T426V the corresponding fragments were ligated into expression vector pFA/CMV (Stratagene) containing the DNA binding domain of yeast transcription factor Gal4 (aa 1–147). Reporter plasmid pG5-luc containing five Gal4 DNA binding sites cloned upstream of a minimal promoter element and the firefly luciferase gene was obtained from Promega (Mannheim, Germany). Expression vector pFC/MEK3 for activated MKK3 (S189D/T193E; MKK3DE) under control of CMV promoter was purchased from Stratagene. The full length cDNA of MKK4 cloned in entry vector (pDONOR201) was obtained from the RZPD consortium (Berlin, Germany). The activated form of MKK4 (S292D/T296E; MKK4DE) was generated by site directed mutagenesis and subcloning into the destination vector pTRExDEST30 designed as pTREx/MKK4DE using the Gateway© cloning system (Invitrogen, Darmstadt, Germany). The Renilla expression vector pRL(-mcs) was generated by eliminating the multiple cloning site of pRL null vector supplied from Promega. Construction of expression vector containing HA tagged N-terminal domain of human SREBP-1a-NT was described previously [49]. To generate transgenic mice with liver specific overexpression, an expression plasmid encoding HA-SREBP-1aΔP (S63A, S117A, T426V) or wild type HA-SREBP-1a was constructed. To assure tissue specific expression in liver the expressing vector, based on pBlueskript II KS (Stratagene), was generated by inserting 2kb of albumin enhancer sequence corresponding to the NheI/BamHI enhancer fragment [50] and the mouse albumin promoter (308 to +8) containing all relevant transacting elements [51] into the BamHI site. All necessary inserts were generated by PCR from C57Bl6 mouse genomic DNA. Subsequently a polyA cassette was inserted via EcoRV/KpnI sites. HA-SREBP-1a-ΔP or HA-SREBP-1a was cloned into this vector construct using the reconstituted BamHI/EcoRV sites. The SREBP-1a expression cassette was released by BssHI restriction, gel purified by using 0.7% agarose gel and QIAquick gel extraction kit (Qiagen, Hilden Germany). The sequences of all constructs were confirmed by sequence analyses using a DNA sequencer model 3100 (Applied Biosystems, Darmstadt, Germany).

### Fusion protein production and protein kinase assay

GST-SREBP-1a-NT or mutated GST-SREBP-1a-NT (S63A, S98A, T105V, S117A, T426V and S63A/T426V) fusion proteins were by expressed in E. coli strain BL21-codon plus (DE3)-RP (Stratagene) and purified according to the manufacturer's recommendations (GE Healthcare, Freiburg, Germany). Phosphorylation of 10 µg GST-SREBP-1a-NT fusion protein or 0.5 µg PHAS by MAPK JNK1, JNK2, p38α, p38β and p38γ (Upstate Biotechnology, Heidelberg, Germany) were performed using activated GST-JNK1, -JNK2, -p38α, -p38β or -p38γ fusion protein (40 ng/µg substrate) in kinase buffer (25 mM Tris/HCl, pH 7.5, 6.25 mM ß-glycerophosphate, 1.25 mM EGTA, 0.25 mM sodium-orthovanadate, 1 mM DTT, 10 mM MgCl_2_). The reaction was initiated by addition of 50 µM [γ^32^P] ATP (10 Ci/mmol) in a final volume of 40 µl kinase buffer. The reaction was terminated after 15 min at 30°C by addition of 5× SDS loading buffer. The phosphorylated proteins were separated by 10% SDS-PAGE and analyzed by autoradiography of the coomassie-stained dried gels.

For electro mobility shift assay 1 µg pcDNA3.1/HisA-SREBP-1a-NT was *in vitro* transcribed and translated into His-tagged protein by TNT T7/T3 coupled reticulocyte lysate system in a final volume of 50 µl according to manufacturer's recommendations (Promega). The His-tagged SREBP-1a-NT was purified using Ni-NTA magnetic agarose beads (Qiagen). For phosphorylation 200 ng of isolated protein was incubated with recombinant activated JNK1 or p38α (40 ng/µg substrate) in kinase buffer according to supplier's instruction manual (Upstate Biotechnology). To control for phosphorylation efficiency, kinase reaction was performed with an aliquot of His-SREBP-1a-NT using additionally 10 µCi [γ^32^P] ATP.

### Peptide-map analyses by High Performance Liquid Chromatography (HPLC)

10 µg of GST-SREBP-1a-NT or mutated GST-SREBP-1a-NT fusion proteins phosphorylated by JNK1, JNK2, p38α, p38β or p38γ as described above were separated on a 10% SDS-PAGE and phosphorylated proteins were in gel digested by trypsin (Promega) over night. The peptides were eluted with 50 mM ammoniumcarbonate and separated on an anion exchange column (Nucleogel SAX 1000-8/46, 50 mm×4.6 mm, Machery & Nagel, Dueren, Germany) using Beckman gold solvent delivery system (Beckman Coulter, Krefeld, Germany). The HPLC flow rate was 0.5 ml/min. After injection of 1 ml sample the peptides were eluted beginning at 100% buffer A (20 mM NH_4_CH_2_COOH pH 7.0) and 0% of buffer B (0.5 M KH_2_PO_4_ pH 4.0). Ratios of B were increased to 10% in 40 min and from 10% to 50% in 75 min. Fractions of 0.5 ml were collected. Cerenkov counting in scintillations counter (Beckman Coulter) determined the relative amount of radiolabeled peptides.

### Identification of p38 MAPK phosphorylation sites by reversed phase HPLC and Matrix Assisted Laser Desorption Ionization Mass Spectrometry (MALDI-MS)

Reversed phase and MALDI-MS analyzes of GST-SREBP-1a-NT fusion proteins phosphorylated by p38α (40 ng/µg substrate) with 250 µM ATP (specific activity of 100 cpm/pmol; Cerenkov) as described above were performed essentially as described [6], [9]. In brief, proteins were separated by SDS-PAGE and phosphorylated GST-SREBP-1a-NT was trypsin digested. Peptides were subjected to reversed phase HPLC and further separated on C_18_-reversed phase column. Fractions containing radiolabeled peptides were subjected to Edman degradation and MALDI-TOF (time of flight) mass spectrometry. Positive ion mass spectra were acquired in a linear and reflector mode using an acceleration voltage of 20,000 V. In addition to manual interpretation of post source decay (PSD) fragment ion spectra the SEQUEST™ algorithm was used to identify the peptide phosphorylation sites. For database search the OWL sequence database (University of Leeds) was used.

### Electrophoretic mobility shift assay (EMSA)

Protein/DNA binding reaction was performed as described with radiolabelled double-stranded oligonucleotide containing the LDLR promoter consensus sre-1 fragment and ADD1/SREBP-1c consensus E-box fragment [17], [19]. The samples were separated on native 5% (w/v) polyacrylamide gels. EMSA was analyzed by autoradiography of the dried gel. Equal loading was confirmed by western blot analysis of 20 µl protein mixture.

### Dual Luciferase Reporter (DLR) gene assay

HepG2 cells (American Tissue Type Collection, HB-8065) were maintained in standard medium (RPMI-1640, 10% (v/v) fetal calf serum (FCS) and antibiotics (Invitrogen). Promoter reporter gene analyses were performed as described [16], [19]. Cell suspension (2×10^5^ cells/well) was mixed with reporter plasmid pG5-luc (0.5 µg/well), with pFA/Gal4-SREBP-1a-NT, pFA/Gal4-SREBP-1a-NT S117A, pFA/Gal4-SREBP-1a-NT S63A/T426V, pFA/Gal4-SREBP-1a-NT S63A/S117A/T426V or pFA/CMV vector (25 ng/well), with pFC/MEK3, pTREx/MKK4 or pFC vector (25 ng/well) and pRL(-mcs) (0.1 µg/well) for control of transfection efficiency, respectively. Then plasmids were transferred to an electroporation cuvette and electroporated. Before seeding on six-well plates cell suspension was diluted with standard medium. On day 1 after transfection cells were cultured in lipid deficient medium (RPMI-1640) containing, 0.5% lipoprotein deficient serum (LPDS) (Sigma-Aldrich, Taufkirchen, Germany)) for 16 h and treated as indicated below. Cell lyses and measurements of firefly as well as renilla luciferase activities of cell extracts (20 µl/probe) were performed according to supplier's instructions (Promega).

### Preparation of cell extracts for western blot analyses

To detect precursor and nuclear SREBP-1 in liver and HA-SREBP-1aΔP or HA-SREBP-1a in liver and other tissues of the transgenic animals at protein level, 10 mg of snap frozen tissue samples (liver, pancreas, heart, small intestine, skeletal muscle) were pottered (10 sec) in 250 µl hypotonic lysis buffer (10 mM Hepes/KOH, pH 7.9, 20 mM KCl, 1.5 mM MgCl_2_, 0.5 mM DTT, 1 mM EDTA, 1 mM EGTA) supplemented with protease inhibitor cocktail (50 µg/ml ALLN, 0.5 mM PMSF, 10 µg/ml leupeptin, 2 µg/ml aprotinin, 0.5 mM benzamidine) and phosphatase inhibitor cocktail (2 mM Na_3_VO_4_, 5 mM KF, 20 mM ß-glycerophosphate). Samples were lysed by shearing 10 times through a 25-gauge needle and centrifuged (2,000× g, 10 min; 4°C). The resulting nuclear pellet was resuspended in hypertonic buffer (20 mM Hepes/KOH, pH 7.9, 400 mM NaCl, 25 mM KCl, 1.5 mM MgCl_2_, 1 mM EDTA, 1 mM EGTA, 20% glycerol (v/v)) supplemented with protease inhibitor cocktail and phosphatase inhibitor cocktail and incubated on a thermomixer (200 rpm, 30 min; 4°C). The extracts were centrifuged (20,000× g, 30 min; 4°C) and aliquots of supernatant, used as nuclear extract, were frozen in liquid nitrogen and stored at −80°C. Protein extracts were resolved on 7.5% SDS-PAGE and electroblotted to nitrocellulose membrane (Hybond™ ECL, GE Healthcare, Munich, Germany) blocked and probed with mouse monoclonal antibody against HA peptide (YPYDVPDYA) conjugated with peroxidase (clone 3F10, 1∶5000, Roche, Mannheim, Germany). A polyclonal SREBP-1 antibody (K10, sc-367, 1∶5000, SantaCruz) was used to determine SREBP-1 precursor protein and the polyclonal SREBP-1 antibody (H160, sc-8984, 1∶5000, SantaCruz) was used to determine the complete nuclear SREBP-1 contends. Visualization was performed with ECL™ plus western blotting detection reagents according to manufacturer's instructions (GE Healthcare, Munich, Germany) and a Versa Doc instrument (BioRad, Munich, Germany).

### Generation of transgenic mice and genotyping

To generate transgenic animals approximately 450 fertilized C57Bl6 oocytes were microinjected with alb-HA-SREBP-1aΔP or alb-HA-SREBP-1a construct and placed into eight foster mice. Of the offspring 5 founders were tested positive for the HA-SREBP-1aΔP and the HA-SREBP-1a construct, respectively. To screen the presence of transgene ear biopsies were taken of the animals at weaning and genomic DNA was extracted with a tissue kit (Qiagen) according to manufactures instructions. Routinely 50 ng DNA was used for PCR with albumin promoter (5′-ATGCGAGGTAAGTAT-3′) and SREBP-1a-NT (5′-TAGGCCAGGGAACTGACTG-3′) specific primers. The presence of the constructs was determined with a DNA 1000 kit on 2100 Bioanalyzer according to manufactures instructions (Agilent, Boeblingen, Germany).

### Animals, phenotypic and metabolic indices

C57Bl6, alb-SREBP-1aΔP and alb-SREBP-1a mice were bred and maintained in our animal facility in a regular 12 h light/dark cycle under constant temperature and humidity (22°C±1°C, 50%±5% humidity). For environmental enrichment the cages were supplemented with nesting material happi-mat® (Scanbur BK, Karlslunde, Denmark) and a mouse house (Techniplast, Hohenpeißenberg, Germany). At the age of six weeks 12 male littermates of each genotype were kept in colonies of four per standard Macrolon III cage (Ebeco, Castrop-Rauxel, Germany). Mice were fed *ad libitum* with standard laboratory diet (13.7 mJ/kg, 53% carbohydrate, 36% protein, 11% fat (Ssniff, Soest, Germany) and they had free access to water. Weekly the animals were controlled for weight gain; food or water intake was also monitored. At the age of 24 weeks alb-SREBP-1aΔP as well as alb-SREBP-1a mice and age matched C57Bl6 mice were sacrificed by CO_2_ asphyxiation. Mice of each genotype of the German Diabetes Center colonies were also transferred to the collaborating Institute for Diabetes Research to verify viability, reproducibility and physiological as well as histological parameters in an independent habitat. The Animal Care Committee of the Universities of Duesseldorf and Hamburg approved animal care and procedure (Approval#50.05-240-35/06 and #93/08).

### Clinical parameters

Blood parameters were measured at 24 weeks of age. Blood glucose was measured with Freestyle™ (Abbott, Wiesbaden, Germany) and triglycerides, cholesterol as well as liver enzymes (ALT, AST) were determined on a Hitachie 912 laboratory automat (Roche, Mannheim, Germany). Serum insulin levels (mU/l) were measured in triplicate by ELISA according to the manufactures recommendation (Mercodia, Uppsala, Sweden). Fatty acid composition of serum or homogenate of liver biopsies were determined on a Packard model 439 gas-liquid chromatograph equipped with a flame ionization detector and a 60 m Hewlett-Packard fused silica capillary column as described [52].

### Relative quantification of RNA by real-time PCR

Total RNA of 10 mg freshly isolated mouse liver biopsies was extracted with RNeasy Mini Spin Kit (Qiagen). Real-time (RT)-PCR was performed in triplicate with cDNA equivalent of 20 ng reversed transcribed RNA (MMVRT, Promega) per reaction.

SREBP-1a and SREBP-1c isoform specific RT-PCR was performed with the conserved probe according to [53] and human specific primers spanning the identical cDNA region [54]. The expression of HA-SREBP-1a and HA-SREBP-1aΔP transgene was confirmed with human specific primers and a primer derived from the HA-tag sequence. Murine SREBP-2 and further genes analyzed were detected with specific primers and probe (Assay on Demand™, Applied Biosystems). RT-PCR was performed under under standardized conditions (2× Universal PCR Mastermix, Applied Biosystems) with an ABI Prism 7000 Sequence Detection System. Data were normalized to 18S RNA content and relative RNA amount was determined according to Fu et al. [55], with ((E_gene_)^−Ctgene^/(E_18S_)^−Ct18S^)*1,000,000 with E = 10^−(1/slope)^.

### Histology

Tissues were resected and fixed in 4% paraformaldehyd/PBS for 24 h and embedded in paraffin with automated standard histological procedures (Excelsior™, Thermo Shandon GmbH, Frankfurt, Germany). On 3 µm deparaffinized sections standard hematoxylin and eosin (HE) staining was performed. Glycogen storage was monitored with PAS staining (Merck, Darmstadt, Germany). Fibers and extra cellular matrix were visualized using the “van Gierson kit” (Merk, Darmstadt, Germany). For oil red O staining, liver was resected and cryoprotected in 30% sucrose 4°C over night. Thereafter tissue was frozen under liquid nitrogen cooled isopentane and stored in liquid nitrogen until proceeding. For cellular lipid detection, 3 µm sections of tissue were prepared and stained with oil-red-O (Sigma). For characterization of cellular histomorphology, fixed slips were stained in Mayer's hemalaun solution (Merck, Darmstadt, Germany) according to the manufacturer's instructions.

### Determination of the cytokine profile in serum and adipocytes

For parallel detection of various cytokines, serum of C57Bl6, alb-SREBP-1aΔP and alb-SREBP-1a mice (n = 6 each) were hybridized to array membranes according to the protocol supplied by the manufacturer (Proteome Profiler™; R&D Systems, Abingdon, UK). Samples of each genotype were analyzed and handeled in parallel troughout the entire procedure. Exposure, data collection and processing of each set of arrays was also performed in parallel (Versa Doc; BioRad). Spots were normalized for internal hybridization positive controls after local background substraction. Normalized data were analysed for genotype specific differences as processed and results per set were used to determine the mean differences in cytokine abundance.

### Statistical analysis

Values are presented as means ± SD. Statistical analysis was performed Student T-test or by 2-way ANOVA calculated with Prism 4.03 (GraphPad Software Inc., San Diego) as indicated.
